# Supplementary material for: Sociodemographic and clinical risk factors for paediatric typical haemolytic uraemic syndrome: retrospective cohort study
Source: BMJ Paediatr Open. 2019 Dec 17;3(1):e000465. doi: 10.1136/bmjpo-2019-000465 (PMC6936988; doi:10.1136/bmjpo-2019-000465)
Supplement: Supplementary data [file bmjpo-2019-000465supp001.pdf]

SUPPLEMENTARY DATA

Supplementary Table 1a: Clinical case definition for HUS

A child (aged <16 years of age) who has:

Acute kidney injury (AKI) defined by oligoanuria and/or elevated creatinine for age

AND

Microangiopathic haemolytic anamia (MAHA) defined by haemoglobin level <10 g/L with fragmented erythrocytes

AND/OR

Thrombocytopenia defined by a platelet count of <130,000 × 109/L

WITHOUT septicaemia, malignant hypertension, chronic uraemia, or primary vascular disease

Elevated creatinine levels differed by age group and were those above the thresholds in Table 1

Supplementary Table 1b: Creatinine level (micromol/L) thresholds by age group

**Age Group**	**Normal Creatinine threshold (micromol/L)**
0-7 days	100
8-14 days	80
15-28 days	55
1m-3 years	40
4-6 years	46
7-9 years	56
10-12 years	60
13-15 years	80

Adams N, *et al. BMJ Paediatrics Open* 2019; 3:e000465. doi: 10.1136/bmjpo-2019-000465

Supplementary Table 2: Adjusted and unadjusted regression analysis - Sensitivity analysis excluding travel cases (n subjects=850)

**Variable**	**Category**	**n (%)**	**Unadjusted**		**Adjusted[a]**		**p value**
			**Odds Ratio**	**(95% CI)**	**Odds Ratio**	**(95% CI)**	
**Age group**	<1	55 (6.5)	0.14	(0.03-0.59)	**0.12**	**(0.02-0.62)**	**0.01**
	1-4	400 (47.1)	1.0 (reference)		1.0 (reference)		
	5-9	242 (28.5)	0.61	(0.39-0.94)	**0.45**	**(0.25-0.79)**	**0.005**
	10-15	153 (18.0)	0.32	(0.17-0.60)	**0.17**	**(0.07-0.39)**	**<0.001**
**Sex**	Male	445 (52.4)	1.0 (reference)		1.0 (reference)		
	Female	405 (47.7)	1.31	(0.91-1.90)	1.30	(0.81-2.08)	0.27
**Ethnicity**	White	700 (82.4)	1.0 (reference)		1.0 (reference)		
	Non-White	150 (17.6)	0.41	(0.20-0.83)	**0.27**	**(0.10-0.73)**	**0.01**
**Rurality**	Urban	606 (71.3)	1.0 (reference)		1.0 (reference)		
	Rural	244 (28.7)	1.28	(0.86-1.90)	1.07	(0.62-1.86)	0.80
**IMD Quintile**	1 (least disadvantaged)	196 (23.1)	1.0 (reference)		1.0 (reference)		
	2	184 (21.7)	0.88	(0.50-1.54)	0.75	(0.36-1.53)	0.43
	3	180 (21.2)	1.24	(0.73-2.10)	1.06	(0.54-2.10)	0.86
	4	150 (17.7)	1.07	(0.61-1.90)	1.14	(0.53-2.46)	0.74
	5 (most disadvantaged)	140 (16.5)	0.52	(0.26-1.04)	0.57	(0.23-1.43)	0.23
**Region**	East Midlands	61 (7.2)	0.54	(0.19-1.49)	0.49	(0.14-1.80)	0.29
	East of England	55 (6.5)	0.84	(0.33-2.16)	1.02	(0.30-3.44)	0.98
	London	83 (9.8)	1.0 (reference)		1.0 (reference)		
	North East	71 (8.4)	0.90	(0.38-2.14)	0.59	(0.19-1.81)	0.35
	North West	172 (20.2)	1.13	(0.56-2.25)	1.06	(0.42-2.67)	0.90
	South East	73 (8.6)	1.27	(0.58-2.86)	1.78	(0.57-5.56)	0.32
	South West	110 (12.9)	1.38	(0.66-2.86)	1.14	(0.42-3.11)	0.80
	West Midlands	95 (11.2)	0.52	(0.21-1.26)	0.57	(0.18-1.78)	0.33
	Yorkshire and Humber	130 (15.3)	0.59	(0.27-1.32)	0.53	(0.19-1.47)	0.22
Stx	*Stx*1+2	183 (21.5)	1.0 (reference)		1.0 (reference)		
	*Stx*1	8 (0.9)	4.21	(0.45-39.89)	**24.71**	**(1.86-328.34)**	**0.02**
	*Stx*2	659 (77.5)	6.97	(3.02-16.10)	**6.08**	**(2.32-15.92)**	**<0.001**
**Antibiotics**	No	766 (90.1)	1.0 (reference)		1.0 (reference)		
	Yes	84 (9.9)	10.0	(6.18-16.32)	**10.89**	**(5.65-20.97)**	**<0.001**
**Diarrhea**	No	48 (6.6)	1.0 (reference)		1.0 (reference)		
	Yes	802 (94.4)	9.26	(1.27-67.70)	4.00	(0.48-33.16)	0.20

Adams N, *et al. BMJ Paediatrics Open* 2019; 3:e000465. doi: 10.1136/bmjpo-2019-000465

**Bloody diarrhea**	No	360 (42.4)	1.0 (reference)		1.0 (reference)		
	Yes	490 (57.7)	5.10	(3.10-8.38)	**3.70**	**(2.00-6.85)**	**<0.001**
**Nausea**	No	562 (66.1)	1.0 (reference)		1.0 (reference)		
	Yes	288 (33.9)	1.52	(1.04-2.22)	1.03	(0.60-1.76)	0.92
**Vomiting**	No	465 (54.7)	1.0 (reference)		1.0 (reference)		
	Yes	385 (45.3)	6.50	(4.13-10.23)	**5.25**	**(2.94-9.38)**	**<0.001**
**Abdominal pain**	No	259 (30.5)	1.0 (reference)		1.0 (reference)		
	Yes	591 (69.5)	1.39	(0.91-2.13)	0.73	(0.39-1.36)	0.32
**Fever**	No	569 (66.9)	1.0 (reference)		1.0 (reference)		
	Yes	281 (33.1)	1.71	(1.17-2.50)	1.28	(0.79-2.09)	0.32

aAdjusted for all other covariates in the model; s*tx* – shiga toxin gene

Supplementary Table 3: Adjusted and unadjusted regression analysis - Sensitivity analysis excluding ethnicity variable (n subjects=989)

Variable	Category	n (%)	Unadjusted		Adjusted[a]		p value
			**Odds Ratio**	**(95% CI)**	**Odds Ratio**	**(95% CI)**	
**Age group**	<1	67 (6.8)	0.19	(0.06-0.62)	**0.24**	**(0.06-0.92)**	**0.04**
	1-4	456 (46.1)	1.0 (reference)		1.0 (reference)		
	5-9	274 (27.7)	0.62	(0.40-0.94)	**0.45**	**(0.26-0.76)**	**0.003**
	10-15	192 (19.4)	0.34	(0.19-0.61)	**0.22**	**(0.11-0.47)**	**<0.001**
**Sex**	Male	513 (51.9)	1.0 (reference)		1.0 (reference)		
	Female	476 (48.1)	1.37	(0.96-1.96)	1.40	(0.91-2.16)	0.13
**Travel**	No	850 (86.0)	1.0 (reference)		1.0 (reference)		
	Yes	139 (14.0)	0.46	(0.24-0.88)	0.58	(0.26-1.28)	0.18
**Rurality**	Urban	719 (72.7)	1.0 (reference)		1.0 (reference)		
	Rural	270 (27.3)	1.21	(0.82-1.77)	0.97	(0.58-1.62)	0.90
**IMD Quintile**	1 (least disadvantaged)	231 (23.4)	1.0 (reference)		1.0 (reference)		
	2	210 (21.2)	0.83	(0.48-1.42)	0.65	(0.33-1.27)	0.21
	3	204 (20.6)	1.28	(0.77-2.12)	0.98	(0.51-1.79)	0.89
	4	170 (17.2)	1.10	(0.64-1.89)	0.96	(0.48-1.94)	0.91
	5 (most disadvantaged)	174 (17.6)	0.57	(0.30-1.06)	**0.41**	**(0.19-0.90)**	**0.03**
**Region**	East Midlands	72 (7.3)	0.62	(0.24-1.59)	0.65	(0.21-2.02)	0.45
	East of England	66 (6.7)	1.03	(0.44-2.42)	1.37	(0.47-4.00)	0.56
	London	108 (10.9)	1.0 (reference)		1.0 (reference)		
	North East	76 (7.7)	1.19	(0.53-2.64)	1.02	(0.38-2.71)	0.97
	North West	185 (18.7)	1.20	(0.63-2.31)	1.29	(0.57-2.90)	0.54
	South East	107 (10.8)	1.09	(0.52-2.28)	1.74	(0.65-4.63)	0.27
	South West	127 (12.8)	1.48	(0.75-2.93)	1.64	(0.68-3.99)	0.27
	West Midlands	104 (10.5)	0.54	(0.23-1.29)	0.67	(0.24-1.86)	0.44
	Yorkshire and Humber	144 (14.6)	0.62	(0.29-1.33)	0.60	(0.24-1.52)	0.28
Stx	*Stx*1+2	226 (22.9)	1.0 (reference)		1.0 (reference)		
	*Stx*1	18 (1.8)	1.84	(0.21-15.84)	5.34	(0.54-52.82)	0.15
	*Stx*2	745 (75.3)	6.99	(3.22-15.17)	**5.76**	**(2.43-13.67)**	**<0.001**
**Antibiotics**	No	887 (89.7)	1.0 (reference)		1.0 (reference)		
	Yes	102 (10.3)	8.54	(5.48-13.30)	**7.46**	**(4.27-13.03)**	**<0.001**
**Diarrhea**	No	49 (5.0)	1.0 (reference)		1.0 (reference)		
	Yes	940 (95.0)	8.61	(1.18-62.89)	4.09	(0.51-32.47)	0.18

Adams N, *et al. BMJ Paediatrics Open* 2019; 3:e000465. doi: 10.1136/bmjpo-2019-000465

**Bloody diarrhea**	No	440 (44.5)	1.0 (reference)		1.0 (reference)		
	Yes	549 (55.5)	4.85	(3.07-7.67)	**3.74**	**(2.15-6.49)**	**<0.001**
**Nausea**	No	653 (66.0)	1.0 (reference)		1.0 (reference)		
	Yes	336 (34.0)	1.52	(1.06-2.18)	1.11	(0.67-1.83)	0.69
**Vomiting**	No	549 (55.5)	1.0 (reference)		1.0 (reference)		
	Yes	440 (44.5)	6.05	(3.95-9.26)	**4.38**	**(2.59-7.40)**	**<0.001**
**Abdominal pain**	No	309 (31.2)	1.0 (reference)		1.0 (reference)		
	Yes	680 (68.8)	1.49	(0.99-2.25)	0.83	(0.47-1.46)	0.52
**Fever**	No	657 (66.4)	1.0 (reference)		1.0 (reference)		
	Yes	332 (33.6)	1.50	(1.05-2.16)	1.04	(0.66-1.63)	0.86

aAdjusted for all other covariates in the model; s*tx* – shiga toxin gene

Adams N, *et al. BMJ Paediatrics Open* 2019; 3:e000465. doi: 10.1136/bmjpo-2019-000465

Supplementary Figure 1: Selection of participants to HUS Cohort Study

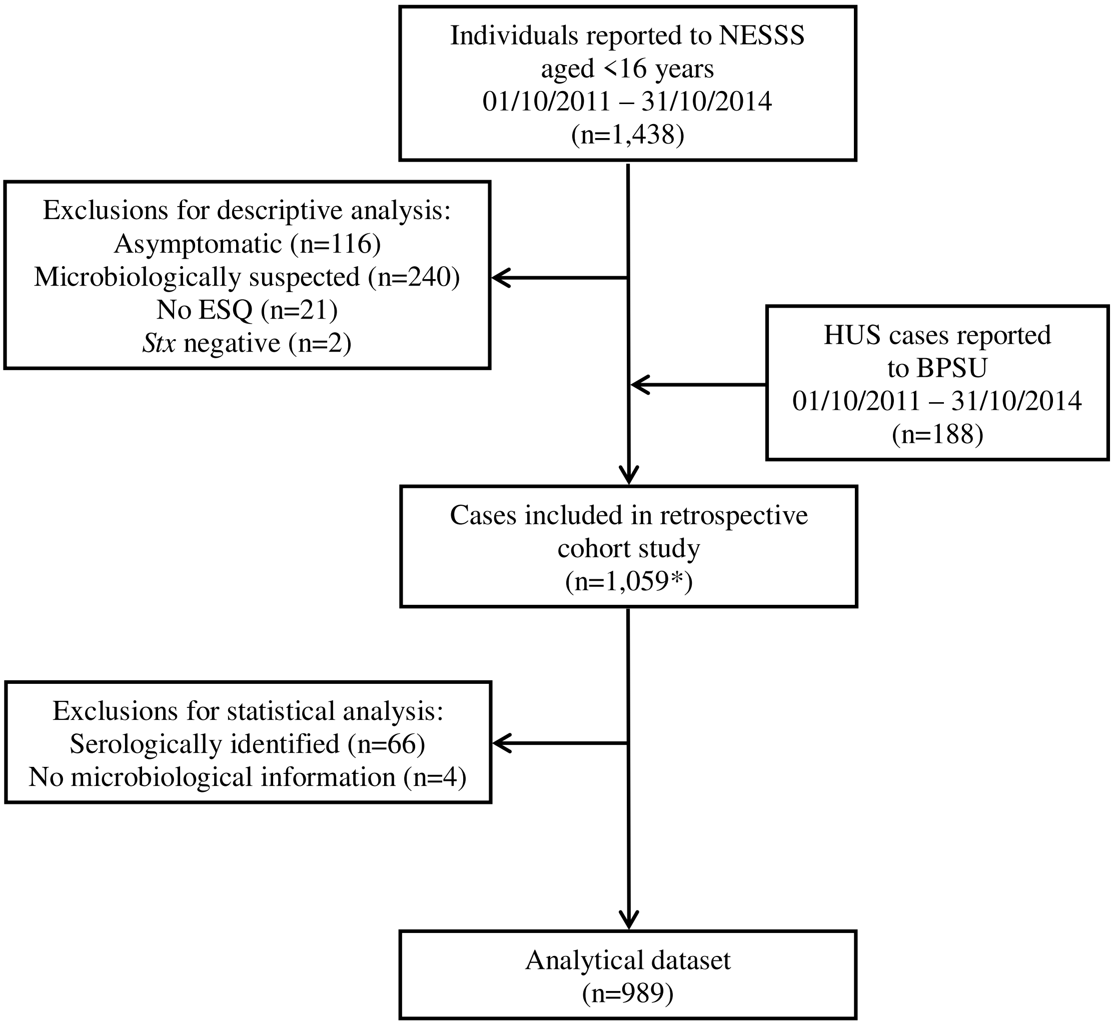

*An additional 19 HUS cases not reported to BPSU were identified in NESSS; NESSS – National Enhanced Surveillance System for STEC; HUS – haemolytic uraemic syndrome; BPSU – British Paediatric Surveillance Unit; ESQ – enhanced surveillance questionnaire

Adams N, *et al. BMJ Paediatrics Open* 2019; 3:e000465. doi: 10.1136/bmjpo-2019-000465
