# Supplementary material for: Sociodemographic and clinical risk factors for paediatric typical haemolytic uraemic syndrome: retrospective cohort study
Source: BMJ Paediatr Open. 2019 Dec 17;3(1):e000465. doi: 10.1136/bmjpo-2019-000465 (PMC6936988; doi:10.1136/bmjpo-2019-000465)
Supplement: Author's manuscript [file bmjpo-2019-000465.draft_revisions.pdf]

BMJ Paediatrics Open

BMJ Paediatrics Open is committed to open peer review. As part of this commitment we make the peer review history of every article we publish publicly available.

When an article is published we post the peer reviewers' comments and the authors' responses online. We also post the versions of the paper that were used during peer review. These are the versions that the peer review comments apply to.

The versions of the paper that follow are the versions that were submitted during the peer review process. They are not the versions of record or the final published versions. They should not be cited or distributed as the published version of this manuscript.

BMJ Paediatrics Open is an open access journal and the full, final, typeset and author-corrected version of record of the manuscript is available on our site with no access controls, subscription charges or pay-per-view fees (http://bmjpaedsopen.bmj.com).

If you have any questions on BMJ Paediatrics Open's open peer review process please email info.bmjpo@bmj.com

BMJ Paediatrics Open

Sociodemographic and clinical risk-factors for paediatric typical haemolytic uraemic syndrome

Journal:	*BMJ Paediatrics Open*
Manuscript ID	bmjpo-2019-000465
Article Type:	Original article
Date Submitted by the Author:	12-Feb-2019
Complete List of Authors:	Adams, Natalie; NIHR Health Protection Research Unit in Gastrointestinal Infections; Public Health England, National Infection Service<br>Byrne, Lisa; Public Health England, National Infection Service<br>Rose, Tanith; NIHR Health Protection Research Unit in Gastrointestinal Infections; University of Liverpool, Department of Public Health and Policy<br>Adak, Bob; NIHR Health Protection Research Unit in Gastrointestinal Infections<br>Jenkins, Claire; Public Health England, National Infection Service; NIHR Health Protection Research Unit in Gastrointestinal Infections<br>Charlett, Andre; Public Health England, National Infection Service<br>Violato, Mara; NIHR Health Protection Research Unit in Gastrointestinal Infections; University of Oxford, Health Economics Research Centre<br>O'Brien, Sarah; NIHR Health Protection Research Unit in Gastrointestinal Infections; University of Liverpool, Department of Public Health and Policy<br>Whitehead, Margaret; NIHR Health Protection Research Unit in Gastrointestinal Infections; University of Liverpool, Department of Public Health and Policy<br>Barr, Benjamin; NIHR Health Protection Research Unit in Gastrointestinal Infections; University of Liverpool, Department of Public Health and Policy<br>Taylor-Robinson, David; NIHR Health Protection Research Unit in Gastrointestinal Infections; University of Liverpool, Department of Public Health and Policy<br>Hawker, Jeremy; NIHR Health Protection Research Unit in Gastrointestinal Infections; Public Health England, National Infection Service
Keywords:	Epidemiology, Infectious Diseases

SCHOLARONE™
Manuscripts

Title Page

Title: Sociodemographic and clinical risk-factors for paediatric typical haemolytic uraemic syndrome

Authors:

Natalie L. Adams*[1,2,3,], Lisa Byrne[1,3], Tanith C. Rose[1,2], Goutam K. Adak[1], Claire Jenkins[1,3], Andre Charlett[3], Mara Violato[1,4], Sarah J. O'Brien[1,2], Margaret Whitehead[1,2], Benjamin Barr[1,2], David Taylor-Robinson[1,2±] and Jeremy Hawker[1,3±]

*Corresponding author

± Joint senior authors

Affiliations:

[1]NIHR Health Protection Research Unit in Gastrointestinal Infections, UK

[2]Department of Public Health and Policy, University of Liverpool, UK

[3]National Infection Service, Public Health England, UK

[4]Health Economics Research Centre, University of Oxford, Oxford, UK

Disclaimers:

The research was funded by the National Institute for Health Research Health Protection Research Unit (NIHR HPRU) in Gastrointestinal Infections at University of Liverpool in partnership with Public Health England (PHE), in collaboration with University of East Anglia, University of Oxford and the Quadram Institute. Natalie Adams is based at the University of Liverpool and Public Health England. The views expressed are those of the

Confidential: For Review Only

author(s) and not necessarily those of the NHS, the NIHR, the Department of Health or Public Health England.

Running title:

Paediatric HUS in England 2011-2014

Corresponding author contact information:

Dr Natalie L Adams, Gastrointestinal Infections Department, National Infection Service, Public Health England, 61 Colindale Ave, Colindale NW9 5EQ, UK

Natalie.Adams@phe.gov.uk

Word count (abstract): 243/250

Word count (text): 2438/2500

References: 31/40

Confidential: For Review Only

ABSTRACT

Objectives

Haemolytic Uraemic Syndrome (HUS) following Shiga toxin-producing *Escherichia coli* (STEC) infection is the commonest cause of acute renal failure among children in the United Kingdom. This study explored differential progression from STEC to HUS by social, demographic and clinical risk factors.

Methods

We undertook a retrospective cohort study linking two datasets. We extracted data on paediatric STEC and HUS cases identified in the Public Health England National Enhanced Surveillance System for STEC and British Paediatric Surveillance Unit HUS surveillance from October 1 2011 to October 31 2014. Using logistic regression, we estimated the odds of HUS progression by risk factors.

Results

1059 paediatric STEC cases were included in the study, of which 207 (19.55%, 95% CI 17-22%) developed HUS. In the fully-adjusted model, the odds of progression to HUS were highest in those aged 1-4 (OR 4.93, 95% CI 2.30-10.56, compared to 10-15 years), were infected with an stx2-only strain (OR 5.92, 95%CI 2.49-14.10), were prescribed antibiotics (OR 8.46, 95% CI 4.71-15.18), had bloody diarrhoea (OR 3.56, 95%CI 2.04-6.24) or vomiting (OR 4.47, 95%CI 2.62-7.63), but there was no association with progression to HUS by socioeconomic circumstances or rurality.

Conclusion

Confidential: For Review Only

Combining data from an active clinical surveillance system for HUS with the national enhanced STEC surveillance system suggests that 20% of diagnosed paediatric STEC infections in England resulted in HUS No relationship was found with socioeconomic status or rurality of cases, but differences were demonstrated by age, stx type and presenting symptoms.

Keywords: Health inequalities; Shiga-toxin producing *Escherichia* coli; STEC; Haemolytic Uraemic Syndrome; HUS

Confidential: For Review Only

INTRODUCTION

Haemolytic uraemic syndrome (HUS) is a rare but serious complication of infection with Shiga toxin-producing *Escherichia coli* (STEC), affecting the blood, kidneys and, in the most severe cases, the central nervous system. Children and the elderly are considered to be the most susceptible age groups and HUS is recognised as the most common cause of acute renal failure among children in the United Kingdom (1). Strains of STEC encoding s*tx2* toxin genes are more often associated with HUS than other strains (1-6).

It is estimated that progression to HUS following STEC infection could be as high as 15% in young children (2, 7). Several studies have suggested that development of HUS varies by some demographic characteristics; higher incidence of HUS has been documented in children (particularly aged 1-4), females (particularly aged over 10 years) and in those of White ethnicity (2, 6, 8-13); however few have documented progression to HUS by other demographic characteristics. This study aims to investigate the relationship between demographic factors, STEC infection and subsequent development of HUS in a well-characterised paediatric population in England with high case-ascertainment.

METHODS

Data, setting and source

We undertook a retrospective cohort study linking two data sources; the Public Health England (PHE) National Enhanced Surveillance System for STEC (NESSS) and the British Paediatric Surveillance Unit (BPSU) HUS Study in conjunction with PHE. Firstly, we extracted data on STEC cases aged 0-15 years (inclusive) identified in NESSS during the period of the BPSU HUS Study (October 1 2011 to October 31 2014). All laboratory-confirmed STEC cases in England are reported by NHS laboratories to Public Health England staff who collect standardised data through an enhanced surveillance questionnaire

Confidential: For Review Only

(ESQ) as part of their public health response: this standardised dataset is collated centrally in NESS for further validation and analysis. The ESQ collects detailed information on patient demographics, symptoms, food and water exposures and UK and non-UK travel during the exposure period (the week prior to illness onset). When a presumptive STEC is identified at the local laboratory or a case of HUS is identified, specimens are sent to PHE Gastrointestinal Bacteria Reference Unit (GBRU) for testing and patient ESQs are linked to microbiological results. This surveillance system is described in detail elsewhere (2).

Secondly, we extracted clinical data on paediatric (<16 years) HUS cases, collected by the BPSU HUS Study, an active surveillance system requiring regular returns from clinicians. Within this study, data were captured using a standardised questionnaire administered to paediatricians collecting information on; case demography; treatment history; microbiological investigations; clinical parameters of illness; clinical management of illness and status of the case at the time of data capture. Cases in the BPSU dataset were linked on National Health Service (NHS) number to those in the NESSS dataset to create a retrospective cohort.

For statistical analysis, cases for whom no microbiological information was available (n=4) and cases identified via serological testing only (n=66) were excluded in order to assess the role of *stx* subtype. Due to missing data for the ethnicity variable (19.1%), we used multiple imputation using chained equations to impute values where ethnicity (white/non-white) was missing for statistical analysis. Fifty imputed datasets were generated. The distribution of ethnicity by age, sex and region was assessed to check the missing at random (MAR) assumption. There was no difference in missing ethnicity by sex, however there were some differences by age group (57.3% of cases with missing ethnicity were in the 1-4 age group; n=114/199) and region (31.2% of cases with missing ethnicity were in London; n=62/199);

https://mc.manuscriptcentral.com/bmjpo

these were not regarded as problematic however as, given the observed data for other variables, the missing data were considered independent.

Ethics

Ethical approval was originally obtained for the main study (Ref: 11/LO/1412). As of October 2010, HUS is a statutory reportable condition and this study falls under the existing Health Protection Agency (now Public Health England) permissions under Section 251 of the NHS Act 2006. In addition, we received a favourable ethical opinion from the South East Coast - Surrey Research Ethics Committee (15/LO/2138) on 1 December 2015 covering the use of this dataset for this study.

Patient involvement

Patients were not directly involved in the design of this study.

Outcome and exposures

The outcome of interest was HUS, determined by the case meeting the BPSU clinical criteria (See supplementary Table 1) (14) or completion of the HUS field in the ESQ. Covariates in the analysis were age group (<1, 1-4, 5-9, 10-15 years); sex (male/female); ethnicity (White/non-White); travel (yes/no); rurality (rural/urban); microbiology (Shiga-toxin (*Stx*)); antibiotic use (yes/no); clinical symptoms (diarrhoea, bloody diarrhoea, nausea, vomiting, abdominal pain, fever) and region of residence. The s*tx* type, the primary STEC virulence factor, was used as the main microbiological variable (15). Where symptoms, travel status and healthcare contact variables were blank or unknown, these were recoded as a negative response. As a proxy for childhood socioeconomic circumstances (SECs) we used a small-area deprivation measure, the Index of Multiple Deprivation 2010 (IMD) (16), assigned to

Confidential: For Review Only

each case based on their postcode and divided into population-level quintiles, with the first quintile representing the least deprived and the fifth quintile representing the most deprived.

Analysis strategy

We explored univariate relationships between progression to HUS and the covariates of interest before fitting a multivariate logistic regression model. All variables were retained in this model in order to control for any potential confounding. Interaction terms between variables (IMD, ethnicity, age and sex) were tested to investigate whether the strength of any relationship was moderated by the inclusion of another variable. Analyses were conducted in Stata 13.1 (Statacorp, Texas).

Robustness tests

We performed multiple sensitivity analyses to test the validity of the main analysis; first by (i) excluding cases that were likely to have a travel-acquired STEC infection (date of onset is within one exposure period, 7 days, of having returned from outside of the UK) and (ii) separately excluding cases with unknown ethnicity to determine whether there were differences in progression to HUS by SECs for children who travelled abroad during their incubation period compared to those who did not or those with ethnicity recorded and those without respectively. Further, to explore the relationship between age and sex in this cohort, we fitted a fractional polynomial prediction plot to detect the best functional form for age (as a continuous variable) and sex by HUS. A likelihood ratio test was performed to best fit.

To further explore potential issues of multicollinearity between IMD and ethnicity a post-hoc matched analysis on ethnicity using conditional logistic regression and penalised logistic regression on the multiply imputed dataset were conducted. The post-hoc matched analysis

https://mc.manuscriptcentral.com/bmjpo

was conducted on a smaller number of variables in order to provide the simplest but most complete model possible.

RESULTS

Descriptive analysis

Of 1059 paediatric STEC cases included in the study, 207 (19.55%, 95% CI 17.27%-22.04%) developed HUS. Progression to HUS varied by age and gender (Table 1), the highest was observed in females aged 1-4 years (26.0%). A higher proportion of progression to HUS was observed in females aged 10-15 years compared to males of the same age (19.3%, 95% CI 12.3-27.9 versus 7.1%, 95% CI 2.9-14.2, p=0.01), and amongst females aged less than 1 year compared to males of the same age, although this was not significant (14.3%, 95% CI 4.0-32.7 versus 4.8%, 95% CI 0.6-16.2, p=0.16). Although progression to HUS was higher in the least disadvantaged quintile (47/245, 19.2%, 95% CI 14.4-24.7) compared with the most disadvantaged quintile (29/189, 15.3%, 95% CI 10.5-21.3) this difference was not statistically significant (p=0.29) and there was no clear pattern across the 5 quintiles (p=0.07), with the highest proportion progressing to HUS in quintile 3.

Multivariable analysis

In the fully adjusted model (Table 2), there were significantly lower odds of HUS amongst <1, 5-9 and 10-15 year olds compared to 1-4 year olds and significantly higher odds of HUS amongst those infected with *stx*2-only strains, those prescribed antibiotics and among those who had experienced bloody diarrhoea or vomiting . The most disadvantaged children had lower odds of progression to HUS compared to the least disadvantaged children (OR 0.57, 95% CI 0.25-1.31) but the difference was not significant. There was no statistically

Confidential: For Review Only

significant difference in risk by rurality or by region. There were no significant interactions identified (data not shown).

The multiple sensitivity analyses conducted to assess the robustness of the findings did not alter the overall conclusions of this research (Supplementary Tables 2-5).

DISCUSSION

In a novel linkage and analysis of two datasets with high case-ascertainment to explore the role of demographic and socioeconomic factors in the development of HUS following STEC infection, we found progression from STEC infection to HUS to be 20% in this paediatric cohort in England. Odds of HUS progression varied by age, stx type, antibiotic exposure and clinical presentation, with children aged 1-4 years infected with stx2-only, with reported antibiotic exposure and presenting with bloody diarrhoea or vomiting at highest risk. Few studies have explored the social patterning of risk factors for STEC (17) or the socio-demographic risk factors associated with progression to HUS, and no such studies have been undertaken in England. We found no relationship between progression to HUS and socioeconomic status in children in this study.

Our study has several strengths. This study captures the progression of HUS in a well-characterised paediatric STEC population. To the best of our knowledge, as confirmed by a prior review of the literature and discussion with national experts, this is the first study to combine a prospective active surveillance system and a multisource national surveillance system to study the risk factors for HUS and as such is likely to have better case-ascertainment of HUS than previous studies and is related to good STEC denominators. Furthermore, this study makes use of one of the largest cohorts of HUS cases. The results of this study are likely to be generalisable to other high-income countries with a similar pattern of STEC infection. Despite this, there are some limitations. It is possible that there is residual

Confidential: For Review Only

confounding that could not be controlled for, such as intrinsic childhood characteristics which may increase differential vulnerability or susceptibility by SEC such as genetic predisposition, co-morbidities, and clinical or treatment characteristics. Further, as an area-level measure of SEC was used, it is possible that it may not have been sensitive enough to detect the effect of socioeconomic inequalities, particularly if individual factors rather than area-level factors have more influence over the risk of acquiring more severe strains of STEC with increased risk of progression to HUS. However, person-to-person spread is an important risk factor for GI infections and therefore community or area-level risk would be an important factor in considering individual risk of infection. Excluding individuals with a serological result only from the statistical analysis may introduce a potential bias leading to an underestimate of HUS incidence, which may be important if there are geographical or host-factors which are linked to severity of illness, although the number of serology-only diagnoses was small. In England, most diagnosed cases of STEC are of serogroup O157, and may therefore not be directly applicable to countries where other, possibly less pathogenic, serogroups predominate. It is possible however, that the risk of progression to HUS could be different in populations exposed to STEC organisms with a lower proportion of stx2-only producing strains, or with a different age distribution of cases. Finally, it was not always possible to determine whether antibiotics had been prescribed during treatment for STEC infection or following a diagnosis of HUS therefore the relevant association should be interpreted with caution.

The finding of 19.5% (95% CI 17-22%) of diagnosed STEC cases progressing to HUS is higher than previous studies, which have estimated the proportion of paediatric cases of STEC O157 progressing to HUS to be 15% (95% CI 11-19%) in females aged 1-4 years in England (2) and 15.3% (95% CI 13-18%) in children aged <5 years in the USA (7). Our study uses data derived from two linked surveillance systems providing high ascertainment of

https://mc.manuscriptcentral.com/bmjpo

Confidential: For Review Only

both STEC and HUS cases which provides a more robust estimate. It is likely that there will also be a bias resulting from ascertainment of STEC cases from laboratory specimens, as milder cases of gastrointestinal infection are less likely to be microbiologically tested, but this will also be true of previous published studies.

Whilst rurality has been reported as an important factor in risk for STEC infection (2, 19), our study suggests that rurality is not a significant driver of progression to HUS. Similarly, despite evidence to suggest that the risk and consequences of GI infections in general are greater for disadvantaged children (20-24) – the finding in our study suggests that lower childhood SEC is unlikely to be a contributor for development of HUS.

Previous studies in England have suggested that children aged 1-4 years, females and white ethnic groups have the highest incidence of STEC infection (2, 25). Our study echoes the findings by Milford et al (6) which demonstrated higher progression to HUS amongst children aged 1-4 years. No overall difference in risk of HUS by sex was identified in our study, a finding echoed in several other previous studies (26-29); this is an area of disagreement in the literature with several studies finding higher risk amongst women (7, 30, 31) although two of these studies finding higher risk in women did not look specifically among children (7, 31). We did find differences in risk by sex within specific age groups, with a greater proportion of progression to HUS amongst girls less than 1 year of age and 10-15 years of age compared to boys of the same age groups (Table 1), although no significant interaction between age and sex could be identified. The reasons for the differential risk by age are currently unclear and call for a deeper understanding of differences in risks and exposures between these groups.

The association between clinical presentation with vomiting and bloody diarrhoea and increased risk of HUS reported in this study has been identified previously (12) and, as such,

https://mc.manuscriptcentral.com/bmjpo

Confidential: For Review Only

the presence of these symptoms particularly in paediatric STEC cases should evoke a high level of clinical suspicion for the potential development of HUS.

Our study quantifies the proportion of paediatric STEC cases progressing to HUS in a well – defined population with high ascertainment. It also quantifies the risk factors associated with progression to HUS in terms of sociodemographic characteristics as well as clinical presentation. Further research is warranted to elucidate the populations at risk of STEC infection and HUS in terms of deprivation, ethnicity, age and sex, in order to better understand whether there are real differences in risk or artefacts of surveillance.

FUNDING

The research was funded by the National Institute for Health Research Health Protection Research Unit (NIHR HPRU) in Gastrointestinal Infections [grant number NIHR HPRU 201210,038] at University of Liverpool in partnership with Public Health England (PHE), in collaboration with University of East Anglia, University of Oxford and the Quadram Institute. Natalie Adams is based at the University of Liverpool and Public Health England. The views expressed are those of the author(s) and not necessarily those of the NHS, the NIHR, the Department of Health and Social Care or Public Health England. David Taylor-Robinson is funded by the MRC on a Clinician Scientist Fellowship (MR/P008577/1). Ben Barr and Tanith Rose were also supported by the National Institute for Health Research (NIHR) Collaboration for Leadership in Health Research and Care (CLAHRC NWC).

CONFLICT OF INTEREST

All authors: No potential conflicts of interest.

ACKNOWLEDGMENTS

Confidential: For Review Only

This work was funded by the National Institute for Health Research Health Protection Research Unit in Gastrointestinal Infections. The authors would like to thank Kirsten Glen and Naomi Launders for their work on the National Enhanced Surveillance System for STEC, Ross Harris for statistical advice and Richard Lynn at the British Paediatric Surveillance Unit for facilitating the BPSU HUS study.

CONFLICT OF INTEREST

The authors declare that they have no conflicts of interest.

Confidential: For Review Only

WHAT IS ALREADY KNOWN ON THIS TOPIC

- HUS is recognised as the most common cause of acute renal failure among children in the United Kingdom.
- It is estimated that progression to HUS following STEC infection could be as high as 15% in young children.
- Several studies have suggested that development of HUS varies by demographic characteristics however few have documented progression to HUS by demographic characteristics.

WHAT THIS STUDY ADDS

- This is a large paediatric cohort including reliable health outcome measures.
- A fifth of paediatric STEC cases developed the serious complication of haemolytic uraemic syndrome in England.
- This figure is higher than previously reported in England, and varied by demographic and clinical, but not socioeconomic, factors.

Confidential: For Review Only

REFERENCES

1. Lynn RM, O'Brien SJ, Taylor CM, et al. Childhood hemolytic uremic syndrome, United Kingdom and Ireland. Emerging Infectious Diseases. 2005;11(4):590-6. doi:10.3201/eid1104.040833
2. Byrne L, Jenkins C, Launders N, Elson R, Adak GK. The epidemiology, microbiology and clinical impact of Shiga toxin-producing Escherichia coli in England, 2009-2012. Epidemiology and Infection. 2015:1-13. doi:10.1017/S0950268815000746
3. Ethelberg S, Olsen KE, Scheutz F, et al. Virulence factors for hemolytic uremic syndrome, Denmark. Emerging Infectious Diseases. 2004;10(5):842-7. doi:10.3201/eid1005.030576
4. Persson S, Olsen KE, Ethelberg S, Scheutz F. Subtyping method for Escherichia coli shiga toxin (verocytotoxin) 2 variants and correlations to clinical manifestations. J Clin Microbiol. 2007;45(6):2020-4. doi:10.1128/JCM.02591-06
5. Dallman TJ, Ashton PM, Byrne L, et al. Applying phylogenomics to understand the emergence of Shiga-toxin-producing Escherichia coli O157:H7 strains causing severe human disease in the UK. Microbial Genomics. 2015;1(3). doi:doi:10.1099/mgen.0.000029
6. Milford DV, Taylor CM, Guttridge B, Hall SM, Rowe B, Kleanthous H. Haemolytic uraemic syndromes in the British Isles 1985-8: association with verocytotoxin producing Escherichia coli. Part 1: Clinical and epidemiological aspects. Archives of Disease in Childhood. 1990;65(7):716-21.
7. Gould LH, Demma L, Jones TF, et al. Hemolytic uremic syndrome and death in persons with Escherichia coli O157:H7 infection, foodborne diseases active surveillance network sites, 2000-2006. Clinical Infectious Diseases. 2009;49(10):1480-5. doi:10.1086/644621
8. Kinney JS, Gross TP, Porter CC, Rogers MF, Schonberger LB, Hurwitz ES. Hemolytic-uremic syndrome: a population-based study in Washington, DC and Baltimore, Maryland. American Journal of Public Health. 1988;78(1):64-5.
9. Rogers MF, Rutherford GW, Alexander SR, et al. A population-based study of hemolytic-uremic syndrome in Oregon, 1979-1982. American Journal of Epidemiology. 1986;123(1):137-42.
10. Tarr PI, Hickman RO. Hemolytic uremic syndrome epidemiology: a population-based study in King County, Washington, 1971 to 1980. Pediatrics. 1987;80(1):41-5.
11. Bell WR. Thrombotic thrombocytopenic purpura/hemolytic uremic syndrome relapse: frequency, pathogenesis, and meaning. Seminars in hematology. 1997;34(2):134-9.
12. Launders N, Byrne L, Jenkins C, Harker K, Charlett A, Adak GK. Disease severity of Shiga toxin-producing E. coli O157 and factors influencing the development of typical haemolytic uraemic syndrome: a retrospective cohort study, 2009–2012. BMJ open. 2016;6(1). doi:10.1136/bmjopen-2015-009933
13. Elliott EJ, Robins-Browne RM, O'Loughlin EV, et al. Nationwide study of haemolytic uraemic syndrome: clinical, microbiological, and epidemiological features. Arch Dis Child. 2001;85:125-31.
14. Byrne L, on behalf of the BPSU HUS Study Team,. BPSU HUS Study Report. 2017.
15. Ethelberg S, Olsen KE, Scheutz F, et al. Virulence factors for hemolytic uremic syndrome, Denmark. Emerg Infect Dis. 2004;10(5):842-7. doi:10.3201/eid1005.030576
16. Department for Communities and Local Government. English Indices of Deprivation 2010. Department for Communities and Local Government. 2011. https://www.gov.uk/government/uploads/system/uploads/attachment_data/file/6871/1871208.pdf. Accessed 16/09/2016.

Confidential: For Review Only

17. Bentancor AB, Ameal LA, Calvino MF, Martinez MC, Miccio L, Degregorio OJ. Risk factors for Shiga toxin-producing Escherichia coli infections in preadolescent schoolchildren in Buenos Aires, Argentina. Journal of Infection in Developing Countries. 2012;6(5):378-86.
18. Byrne L, Vanstone GL, Perry NT, et al. Epidemiology and microbiology of Shiga toxin-producing Escherichia coli other than serogroup O157 in England, 2009-2013. Journal of medical microbiology. 2014;63(Pt 9):1181-8. doi:10.1099/jmm.0.075895-0
19. Chang M, Groseclose SL, Zaidi AA, Braden CR. An ecological analysis of sociodemographic factors associated with the incidence of salmonellosis, shigellosis, and E. coli O157:H7 infections in US counties. Epidemiology and infection. 2009;137(6):810-20. doi:10.1017/s0950268808001477
20. Olowokure B, Hawker J, Weinberg J, Gill N, Sufi F. Deprivation and hospital admission for infectious intestinal diseases. Lancet. 1999;353(9155):807-8. doi:10.1016/S0140-6736(99)00611-X
21. Phillips G, Tam CC, Rodrigues LC, Lopman B. Risk factors for symptomatic and asymptomatic norovirus infection in the community. Epidemiology and Infection. 2011;139(11):1676-86. doi:http://dx.doi.org/10.1017/S0950268810002839
22. Pockett RD, Adlard N, Carroll S, Rajoriya F. Paediatric hospital admissions for rotavirus gastroenteritis and infectious gastroenteritis of all causes in England: an analysis of correlation with deprivation. Current medical research and opinion. 2011;27(4):777-84. doi:10.1185/03007995.2011.555757
23. Rose TC, Adams NL, Barr B, et al. Socioeconomic status is associated with symptom severity and sickness absence in people with infectious intestinal disease in the UK. BMC Infect Dis. 2017;17(1):447. doi:10.1186/s12879-017-2551-1
24. Adams NL, Rose TC, Hawker J, et al. Relationship between socioeconomic status and gastrointestinal infections in developed countries: A systematic review and meta-analysis. PloS one. 2018;13(1):e0191633. doi:10.1371/journal.pone.0191633
25. Adams NL, Byrne L, Smith GA, et al. Shiga Toxin-Producing Escherichia coli O157, England and Wales, 1983-2012. Emerging Infectious Diseases. 2016;22(4):590-7. doi:10.3201/eid2204.151485
26. Tserenpuntsag B, Chang H-G, Smith PF, Morse DL. Hemolytic uremic syndrome risk and Escherichia coli O157:H7. Emerging Infectious Diseases. 2005;11(12):1955-7.
27. Bell BP, Griffin PM, Lozano P, Christie DL, Kobayashi JM, Tarr PI. Predictors of hemolytic uremic syndrome in children during a large outbreak of Escherichia coli O157:H7 infections. Pediatrics. 1997;100(1):E12.
28. Rowe PC, Orrbine E, Lior H, et al. Risk of hemolytic uremic syndrome after sporadic Escherichia coli O157:H7 infection: results of a Canadian collaborative study. Investigators of the Canadian Pediatric Kidney Disease Research Center. Journal of Pediatrics. 1998;132(5):777-82.
29. Cimolai N, Basalyga S, Mah DG, Morrison BJ, Carter JE. A continuing assessment of risk factors for the development of Escherichia coli O157:H7-associated hemolytic uremic syndrome. Clinical Nephrology. 1994;42(2):85-9.
30. Rowe PC, Orrbine E, Wells GA, McLaine PN. Epidemiology of hemolytic-uremic syndrome in Canadian children from 1986 to 1988. The Canadian Pediatric Kidney Disease Reference Centre. Journal of Pediatrics. 1991;119(2):218-24.
31. Chang H-GH, Tserenpuntsag B, Kacica M, Smith PF, Morse DL. Hemolytic uremic syndrome incidence in New York. Emerging Infectious Diseases. 2004;10(5):928-31.

Confidential: For Review Only

TABLES

Table 1: Characteristics of cohort participants by HUS status (n=1,059)

		No HUS<br>n (%)	HUS<br>n (%)
**Total**		852 (80.5)	207 (19.6)
**Age group**	<1	64 (91.4)	6 (8.6)
	1-4	370 (76.1)	116 (23.9)
	5-9	239 (80.7)	57 (19.3)
	10-15	179 (86.5)	28 (13.5)
**Sex**	Female	400 (77.5)	116 (22.5)
	Male	452 (83.2)	91 (16.8)
**Age and Sex**	Female <1	24 (85.7)	4 (14.3)
	Female 1-4	171 (74.0)	60 (26.0)
	Female 5-9	117 (79.1)	31 (20.9)
	Female 10-15	88 (80.7)	21 (19.3)
	Male <1	40 (95.2)	2 (4.8)
	Male 1-4	199 (78.0)	56 (22.0)
	Male 5-9	122 (82.4)	26 (17.6)
	Male 10-15	91 (92.9)	7 (7.1)
**Ethnicity**	White	552 (80.5)	134 (19.5)
	Non-white	138 (88.5)	18 (11.5)
	Unknown	162 (74.7)	55 (23.4)
**IMD Quintile**	1 (Least Disadvantaged)	198 (80.8)	47 (19.2)
	2	186 (84.2)	35 (15.8)
	3	166 (75.8)	53 (24.2)
	4	142 (76.8)	43 (23.2)
	5 (Most Disadvantaged)	160 (84.7)	29 (15.3)
**Travel**	Yes	128 (85.3)	22 (14.7)
	No	724 (79.7)	185 (20.4)
**Rurality**	Rural	230 (80.4)	56 (19.6)
	Urban	622 (80.5)	151 (19.5)
**Region**	East Midlands	65 (81.3)	15 (18.8)
	East of England	57 (80.3)	14 (19.7)
	London	93 (81.6)	21 (18.4)
	North East	64 (77.1)	19 (22.9)
	North West	153 (77.7)	44 (22.3)
	South East	92 (78.6)	25 (21.4)
	South West	101 (75.9)	32 (24.1)
	West Midlands	96 (84.2)	18 (15.8)
	Yorkshire and Humber	131 (87.3)	19 (12.7)
Stx	*Stx*1	17 (94.4)	1 (5.6)
	*Stx*2	609 (81.7)	136 (18.3)
	*Stx*1+2	219 (96.9)	7 (3.1)
	Serology	7 (10.6)	59 (89.4)
	Unknown	0 (0.0)	4 (100.0)
**Symptoms**	Diarrhoea	803 (80.3)	197 (19.7)
	Bloody diarrhoea	432 (74.0)	152 (26.0)
	Nausea	278 (75.8)	89 (24.3)
	Vomiting	330 (66.1)	169 (33.9)
	Abdominal pain	574 (78.2)	160 (21.8)

Confidential: For Review Only

	Fever	273 (76.7)	83 (23.3)
**Healthcare contact**	Antibiotics	53 (40.8)	77 (59.2)
	NHS Direct	67 (72.0)	26 (28.0)
	GP	570 (83.7)	111 (16.3)
	A&E	186 (66.9)	92 (33.1)
	Other healthcare contact	98 (74.8)	33 (25.2)
	Hospital	223 (52.4)	203 (47.6)

HUS – haemolytic uraemic syndrome; *stx* – Shiga toxin; NHS Direct – National Health Service telephone advice line, now NHS 111; GP – General Practitioner; A&E – accident and emergency

Confidential: For Review Only

Table 2: Adjusted and unadjusted regression analysis (n=989)

**Variable**	**Category**	**n (%)**	**Unadjusted**		**Adjusted[a]**		**p value**
			**Odds Ratio**	**(95% CI)**	**Odds Ratio**	**(95% CI)**	
**Age group**	<1	67 (6.8)	0.19	(0.06-0.62)	**0.21**	**(0.05-0.82)**	**0.03**
	1-4	456 (46.1)	1.0 (reference)		1.0 (reference)		
	5-9	274 (27.7)	0.62	(0.40-0.94)	**0.43**	**(0.25-0.74)**	**0.002**
	10-15	192 (19.4)	0.34	(0.19-0.61)	**0.20**	**(0.09-0.43)**	**<0.001**
**Sex**	Male	513 (51.9)	1.0 (reference)		1.0 (reference)		
	Female	476 (48.1)	1.37	(0.96-1.96)	1.38	(0.88-2.14)	0.16
**Ethnicity[±]**	White	797 (80.6)	1.0 (reference)		1.0 (reference)		
	Non-White	192 (19.4)	0.39	(0.18-0.81)	**0.28**	**(0.11-0.74)**	**0.01**
**Travel**	No	850 (86.0)	1.0 (reference)		1.0 (reference)		
	Yes	139 (14.0)	0.46	(0.24-0.88)	0.64	(0.28-1.45)	0.28
**Rurality**	Urban	719 (72.7)	1.0 (reference)		1.0 (reference)		
	Rural	270 (27.3)	1.21	(0.82-1.77)	0.88	(0.52-1.48)	0.63
**IMD Quintile**	1 (least disadvantaged)	231 (23.4)	1.0 (reference)		1.0 (reference)		
	2	210 (21.2)	0.83	(0.48-1.42)	0.64	(0.32-1.27)	0.20
	3	204 (20.6)	1.28	(0.77-2.12)	1.01	(0.54-1.91)	0.97
	4	170 (17.2)	1.10	(0.64-1.90)	1.10	(0.54-2.26)	0.79
	5 (most disadvantaged)	174 (17.6)	0.57	(0.30-1.06)	0.57	(0.25-1.31)	0.18
**Region**	East Midlands	72 (7.3)	0.62	(0.24-1.59)	0.59	(0.18-1.92)	0.39
	East of England	66 (6.7)	1.03	(0.44-2.42)	1.12	(0.37-3.37)	0.84
	London	108 (10.9)	1.0 (reference)		1.0 (reference)		
	North East	76 (7.7)	1.19	(0.53-2.64)	0.71	(0.26-1.97)	0.51
	North West	185 (18.7)	1.20	(0.63-2.31)	1.02	(0.44-2.37)	0.97
	South East	107 (10.8)	1.09	(0.52-2.28)	1.31	(0.48-3.63)	0.60
	South West	127 (12.8)	1.48	(0.75-2.93)	1.25	(0.50-3.13)	0.63
	West Midlands	104 (10.5)	0.54	(0.23-1.29)	0.53	(0.18-1.53)	0.24
	Yorkshire and Humber	144 (14.6)	0.62	(0.29-1.33)	0.52	(0.20-1.34)	0.17
Stx	*Stx*1+2	226 (22.9)	1.0 (reference)		1.0 (reference)		
	*Stx*1	18 (1.8)	1.84	(0.21-15.84)	5.53	(0.53-57.42)	0.15
	*Stx*2	745 (75.3)	6.99	(3.22-15.17)	**5.92**	**(2.49-14.10)**	**<0.001**
**Antibiotics**	No	887 (89.7)	1.0 (reference)		1.0 (reference)		
	Yes	102 (10.3)	8.54	(5.48-13.30)	**8.46**	**(4.71-15.18)**	**<0.001**

Confidential: For Review Only

**Diarrhoea**	No	49 (5.0)	1.0 (reference)		1.0 (reference)		
	Yes	940 (95.0)	8.61	(1.18-62.89)	4.04	(0.50-32.59)	0.19
**Bloody diarrhoea**	No	440 (44.5)	1.0 (reference)		1.0 (reference)		
	Yes	549 (55.5)	4.85	(3.07-8.00)	**3.56**	**(2.04-6.24)**	**<0.001**
**Nausea**	No	653 (66.0)	1.0 (reference)		1.0 (reference)		
	Yes	336 (34.0)	1.52	(1.06-2.18)	1.12	(0.67-1.86)	0.66
**Vomiting**	No	549 (55.5)	1.0 (reference)		1.0 (reference)		
	Yes	440 (44.5)	6.05	(3.95-9.26)	**4.47**	**(2.62-7.63)**	**<0.001**
**Abdominal pain**	No	309 (31.2)	1.0 (reference)		1.0 (reference)		
	Yes	680 (68.8)	1.49	(0.99-2.25)	0.82	(0.46-1.46)	0.50
**Fever**	No	657 (66.4)	1.0 (reference)		1.0 (reference)		
	Yes	332 (33.6)	1.50	(1.05-2.16)	1.05	(0.67-1.66)	0.82

[a]Adjusted for all other covariates in the model; *stx* – Shiga toxin gene; [±]Multiply imputed variable

Confidential: For Review Only

SUPPLEMENTARY DATA

Supplementary Table 1a: Clinical case definition for HUS

A child (aged <16 years of age) who has:
Acute kidney injury (AKI) defined by oligoanuria and/or elevated creatinine for age
AND
Microangiopathic haemolytic anamia (MAHA) defined by haemoglobin level <10 g/L with fragmented erythrocytes
AND/OR
Thrombocytopenia defined by a platelet count of <130,000 × 109/L
WITHOUT septicaemia, malignant hypertension, chronic uraemia, or primary vascular disease
Elevated creatinine levels differed by age group and were those above the thresholds in Table 1

Supplementary Table 1b: Creatinine level (micromol/L) thresholds by age group

**Age Group**	**Normal Creatinine threshold (micromol/L)**
0-7 days	100
8-14 days	80
15-28 days	55
1m-3 years	40
4-6 years	46
7-9 years	56
10-12 years	60
13-15 years	80

Confidential: For Review Only

Supplementary Table 2: Adjusted and unadjusted regression analysis - Sensitivity analysis excluding travel cases (n subjects=850)

Variable	Category	n (%)	Unadjusted		Adjusted[a]		p value
			Odds Ratio	(95% CI)	Odds Ratio	(95% CI)	
**Age group**	<1	55 (6.5)	0.14	(0.03-0.59)	**0.12**	**(0.02-0.62)**	**0.01**
	1-4	400 (47.1)	1.0 (reference)		1.0 (reference)		
	5-9	242 (28.5)	0.61	(0.39-0.94)	**0.45**	**(0.25-0.79)**	**0.005**
	10-15	153 (18.0)	0.32	(0.17-0.60)	**0.17**	**(0.07-0.39)**	**<0.001**
**Sex**	Male	445 (52.4)	1.0 (reference)		1.0 (reference)		
	Female	405 (47.7)	1.31	(0.91-1.90)	1.30	(0.81-2.08)	0.27
**Ethnicity**	White	700 (82.4)	1.0 (reference)		1.0 (reference)		
	Non-White	150 (17.6)	0.41	(0.20-0.83)	**0.27**	**(0.10-0.73)**	**0.01**
**Rurality**	Urban	606 (71.3)	1.0 (reference)		1.0 (reference)		
	Rural	244 (28.7)	1.28	(0.86-1.90)	1.07	(0.62-1.86)	0.80
**IMD Quintile**	1 (least disadvantaged)	196 (23.1)	1.0 (reference)		1.0 (reference)		
	2	184 (21.7)	0.88	(0.50-1.54)	0.75	(0.36-1.53)	0.43
	3	180 (21.2)	1.24	(0.73-2.10)	1.06	(0.54-2.10)	0.86
	4	150 (17.7)	1.07	(0.61-1.90)	1.14	(0.53-2.46)	0.74
	5 (most disadvantaged)	140 (16.5)	0.52	(0.26-1.04)	0.57	(0.23-1.43)	0.23
**Region**	East Midlands	61 (7.2)	0.54	(0.19-1.49)	0.49	(0.14-1.80)	0.29
	East of England	55 (6.5)	0.84	(0.33-2.16)	1.02	(0.30-3.44)	0.98
	London	83 (9.8)	1.0 (reference)		1.0 (reference)		
	North East	71 (8.4)	0.90	(0.38-2.14)	0.59	(0.19-1.81)	0.35
	North West	172 (20.2)	1.13	(0.56-2.25)	1.06	(0.42-2.67)	0.90
	South East	73 (8.6)	1.27	(0.58-2.86)	1.78	(0.57-5.56)	0.32
	South West	110 (12.9)	1.38	(0.66-2.86)	1.14	(0.42-3.11)	0.80
	West Midlands	95 (11.2)	0.52	(0.21-1.26)	0.57	(0.18-1.78)	0.33
	Yorkshire and Humber	130 (15.3)	0.59	(0.27-1.32)	0.53	(0.19-1.47)	0.22
Stx	*Stx*1+2	183 (21.5)	1.0 (reference)		1.0 (reference)		
	*Stx*1	8 (0.9)	4.21	(0.45-39.89)	**24.71**	**(1.86-328.34)**	**0.02**
	*Stx*2	659 (77.5)	6.97	(3.02-16.10)	**6.08**	**(2.32-15.92)**	**<0.001**
**Antibiotics**	No	766 (90.1)	1.0 (reference)		1.0 (reference)		
	Yes	84 (9.9)	10.0	(6.18-16.32)	**10.89**	**(5.65-20.97)**	**<0.001**
**Diarrhea**	No	48 (6.6)	1.0 (reference)		1.0 (reference)		
	Yes	802 (94.4)	9.26	(1.27-67.70)	4.00	(0.48-33.16)	0.20

Confidential: For Review Only

**Bloody diarrhea**	No	360 (42.4)	1.0 (reference)		1.0 (reference)		
	Yes	490 (57.7)	5.10	(3.10-8.38)	**3.70**	**(2.00-6.85)**	**<0.001**
**Nausea**	No	562 (66.1)	1.0 (reference)		1.0 (reference)		
	Yes	288 (33.9)	1.52	(1.04-2.22)	1.03	(0.60-1.76)	0.92
**Vomiting**	No	465 (54.7)	1.0 (reference)		1.0 (reference)		
	Yes	385 (45.3)	6.50	(4.13-10.23)	**5.25**	**(2.94-9.38)**	**<0.001**
**Abdominal pain**	No	259 (30.5)	1.0 (reference)		1.0 (reference)		
	Yes	591 (69.5)	1.39	(0.91-2.13)	0.73	(0.39-1.36)	0.32
**Fever**	No	569 (66.9)	1.0 (reference)		1.0 (reference)		
	Yes	281 (33.1)	1.71	(1.17-2.50)	1.28	(0.79-2.09)	0.32

aAdjusted for all other covariates in the model; *stx* – shiga toxin gene

Confidential: For Review Only

Supplementary Table 3: Adjusted and unadjusted regression analysis - Sensitivity analysis excluding ethnicity variable (n subjects=989)

Variable	Category	n (%)	Unadjusted		Adjusted[a]		p value
			Odds Ratio	(95% CI)	Odds Ratio	(95% CI)	
**Age group**	<1	67 (6.8)	0.19	(0.06-0.62)	**0.24**	**(0.06-0.92)**	**0.04**
	1-4	456 (46.1)	1.0 (reference)		1.0 (reference)		
	5-9	274 (27.7)	0.62	(0.40-0.94)	**0.45**	**(0.26-0.76)**	**0.003**
	10-15	192 (19.4)	0.34	(0.19-0.61)	**0.22**	**(0.11-0.47)**	**<0.001**
**Sex**	Male	513 (51.9)	1.0 (reference)		1.0 (reference)		
	Female	476 (48.1)	1.37	(0.96-1.96)	1.40	(0.91-2.16)	0.13
**Travel**	No	850 (86.0)	1.0 (reference)		1.0 (reference)		
	Yes	139 (14.0)	0.46	(0.24-0.88)	0.58	(0.26-1.28)	0.18
**Rurality**	Urban	719 (72.7)	1.0 (reference)		1.0 (reference)		
	Rural	270 (27.3)	1.21	(0.82-1.77)	0.97	(0.58-1.62)	0.90
**IMD Quintile**	1 (least disadvantaged)	231 (23.4)	1.0 (reference)		1.0 (reference)		
	2	210 (21.2)	0.83	(0.48-1.42)	0.65	(0.33-1.27)	0.21
	3	204 (20.6)	1.28	(0.77-2.12)	0.98	(0.51-1.79)	0.89
	4	170 (17.2)	1.10	(0.64-1.89)	0.96	(0.48-1.94)	0.91
	5 (most disadvantaged)	174 (17.6)	0.57	(0.30-1.06)	**0.41**	**(0.19-0.90)**	**0.03**
**Region**	East Midlands	72 (7.3)	0.62	(0.24-1.59)	0.65	(0.21-2.02)	0.45
	East of England	66 (6.7)	1.03	(0.44-2.42)	1.37	(0.47-4.00)	0.56
	London	108 (10.9)	1.0 (reference)		1.0 (reference)		
	North East	76 (7.7)	1.19	(0.53-2.64)	1.02	(0.38-2.71)	0.97
	North West	185 (18.7)	1.20	(0.63-2.31)	1.29	(0.57-2.90)	0.54
	South East	107 (10.8)	1.09	(0.52-2.28)	1.74	(0.65-4.63)	0.27
	South West	127 (12.8)	1.48	(0.75-2.93)	1.64	(0.68-3.99)	0.27
	West Midlands	104 (10.5)	0.54	(0.23-1.29)	0.67	(0.24-1.86)	0.44
	Yorkshire and Humber	144 (14.6)	0.62	(0.29-1.33)	0.60	(0.24-1.52)	0.28
Stx	*Stx*1+2	226 (22.9)	1.0 (reference)		1.0 (reference)		
	*Stx*1	18 (1.8)	1.84	(0.21-15.84)	5.34	(0.54-52.82)	0.15
	*Stx*2	745 (75.3)	6.99	(3.22-15.17)	**5.76**	**(2.43-13.67)**	**<0.001**
**Antibiotics**	No	887 (89.7)	1.0 (reference)		1.0 (reference)		
	Yes	102 (10.3)	8.54	(5.48-13.30)	**7.46**	**(4.27-13.03)**	**<0.001**

Confidential: For Review Only

**Diarrhea**	No	49 (5.0)	1.0 (reference)		1.0 (reference)		
	Yes	940 (95.0)	8.61	(1.18-62.89)	4.09	(0.51-32.47)	0.18
**Bloody diarrhea**	No	440 (44.5)	1.0 (reference)		1.0 (reference)		
	Yes	549 (55.5)	4.85	(3.07-7.67)	**3.74**	**(2.15-6.49)**	**<0.001**
**Nausea**	No	653 (66.0)	1.0 (reference)		1.0 (reference)		
	Yes	336 (34.0)	1.52	(1.06-2.18)	1.11	(0.67-1.83)	0.69
**Vomiting**	No	549 (55.5)	1.0 (reference)		1.0 (reference)		
	Yes	440 (44.5)	6.05	(3.95-9.26)	**4.38**	**(2.59-7.40)**	**<0.001**
**Abdominal pain**	No	309 (31.2)	1.0 (reference)		1.0 (reference)		
	Yes	680 (68.8)	1.49	(0.99-2.25)	0.83	(0.47-1.46)	0.52
**Fever**	No	657 (66.4)	1.0 (reference)		1.0 (reference)		
	Yes	332 (33.6)	1.50	(1.05-2.16)	1.04	(0.66-1.63)	0.86

aAdjusted for all other covariates in the model; *stx* – shiga toxin gene

Confidential: For Review Only

Supplementary Table 4: Comparison between logistic regression model and post-hoc matched analysis on ethnicity

Variable	Category	Main model[a]		Post-hoc matched analysis[a]	
		**Odds Ratio**	**(95% CI)**	**Odds Ratio**	**(95% CI)**
**Age group**	<1	**0.21**	**(0.05-0.82)**	0.40	(0.13-0.90)
	1-4	1.0 (reference)		1.0 (reference)	
	5-9	**0.43**	**(0.25-0.74)**	0.66	(0.31-1.35)
	10-15	**0.20**	**(0.09-0.43)**	0.28	(0.01-1.01)
**Sex**	Male	1.0 (reference)		1.0 (reference)	
	Female	1.38	(0.88-2.14)	1.76	(0.96-3.34)
**Travel**	No	1.0 (reference)		1.0 (reference)	
	Yes	0.64	(0.28-1.45)	0.71	(0.24-1.99)
Stx	*Stx*1+2	1.0 (reference)		1.0 (reference)	
	*Stx*2	**5.92**	**(2.49-14.10)**	**14.37**	**(6.66-67.74)**
**IMD Quintile**	1 (least disadvantaged)	1.0 (reference)		1.0 (reference)	
	2	0.64	(0.32-1.27)	0.93	(0.44-2.15)
	3	1.01	(0.54-1.91)	1.40	(0.63-3.30)
	4	1.10	(0.54-2.26)	1.39	(0.58-3.82)
	5 (most disadvantaged)	0.57	(0.25-1.31)	0.63	(0.23-1.70)

aAdjusted for all other covariates in the model; *stx* – shiga toxin gene

Confidential: For Review Only

Supplementary Table 5: Comparison between logistic regression model and penalized logistic regression model

Variable	Category	Main model[a]		Penalized logistic regression[a]	
		**Odds Ratio**	**(95% CI)**	**Odds Ratio**	**(95% CI)**
**Age group**	<1	**0.21**	**(0.05-0.82)**	**0.21**	**(0.05-0.82)**
	1-4	1.0 (reference)		1.0 (reference)	
	5-9	**0.43**	**(0.25-0.74)**	**0.20**	**(0.09-0.44)**
	10-15	**0.20**	**(0.09-0.43)**	**0.43**	**(0.25-0.74)**
**Sex**	Male	1.0 (reference)		1.0 (reference)	
	Female	1.38	(0.88-2.14)	1.37	(0.88-2.13)
**Ethnicity**	White	1.0 (reference)		1.0 (reference)	
	Non-White	**0.28**	**(0.11-0.74)**	**0.32**	**(0.11-0.96)**
**Travel**	No	1.0 (reference)		1.0 (reference)	
	Yes	0.64	(0.28-1.45)	0.64	(0.28-1.44)
**Rurality**	Urban	1.0 (reference)		1.0 (reference)	
	Rural	0.88	(0.52-1.48)	0.88	(0.52-1.49)
**IMD Quintile**	1 (least disadvantaged)	1.0 (reference)		1.0 (reference)	
	2	0.64	(0.32-1.27)	0.64	(0.32-1.27)
	3	1.01	(0.54-1.91)	1.01	(0.54-1.91)
	4	1.10	(0.54-2.26)	1.09	(0.53-2.23)
	5 (most disadvantaged)	0.57	(0.25-1.31)	0.55	(0.24-1.26)
**Region**	East Midlands	0.59	(0.18-1.92)	0.59	(0.18-1.91)
	East of England	1.12	(0.37-3.37)	1.16	(0.39-3.47)
	London	1.0 (reference)		1.0 (reference)	
	North East	0.71	(0.26-1.97)	0.73	(0.26-2.05)
	North West	1.02	(0.44-2.37)	1.03	(0.44-2.41)
	South East	1.31	(0.48-3.63)	1.34	(0.48-3.73)
	South West	1.25	(0.50-3.13)	1.28	(0.51-3.21)
	West Midlands	0.53	(0.18-1.53)	0.54	(0.91-1.56)
	Yorkshire and Humber	0.52	(0.20-1.34)	0.52	(0.20-1.35)
Stx	*Stx*1+2	1.0 (reference)		1.0 (reference)	
	*Stx*1	5.53	(0.53-57.42)	5.58	(0.54-57.77)
	*Stx*2	**5.92**	**(2.49-14.10)**	**5.89**	**(2.38-14.02)**
**Antibiotics**	No	1.0 (reference)		1.0 (reference)	
	Yes	**8.46**	**(4.71-15.18)**	**8.36**	**(4.65-15.03)**

Confidential: For Review Only

**Diarrhea**	No	1.0 (reference)		1.0 (reference)	
	Yes	4.04	(0.50-32.59)	3.97	(0.49-31.93)
**Bloody diarrhea**	No	1.0 (reference)		1.0 (reference)	
	Yes	**3.56**	**(2.04-6.24)**	**3.54**	**(2.02-6.21)**
**Nausea**	No	1.0 (reference)		1.0 (reference)	
	Yes	1.12	(0.67-1.86)	1.12	(0.67-1.86)
**Vomiting**	No	1.0 (reference)		1.0 (reference)	
	Yes	**4.47**	**(2.62-7.63)**	**4.48**	**(2.63-7.64)**
**Abdominal pain**	No	1.0 (reference)		1.0 (reference)	
	Yes	0.82	(0.46-1.46)	0.81	(0.46-1.45)
**Fever**	No	1.0 (reference)		1.0 (reference)	
	Yes	1.05	(0.67-1.66)	0.81	(0.67-1.66)

aAdjusted for all other covariates in the model; *stx* – shiga toxin gene

Confidential: For Review Only

Supplementary Figure 1: Fractional polynomial prediction plots for age and sex by HUS Status

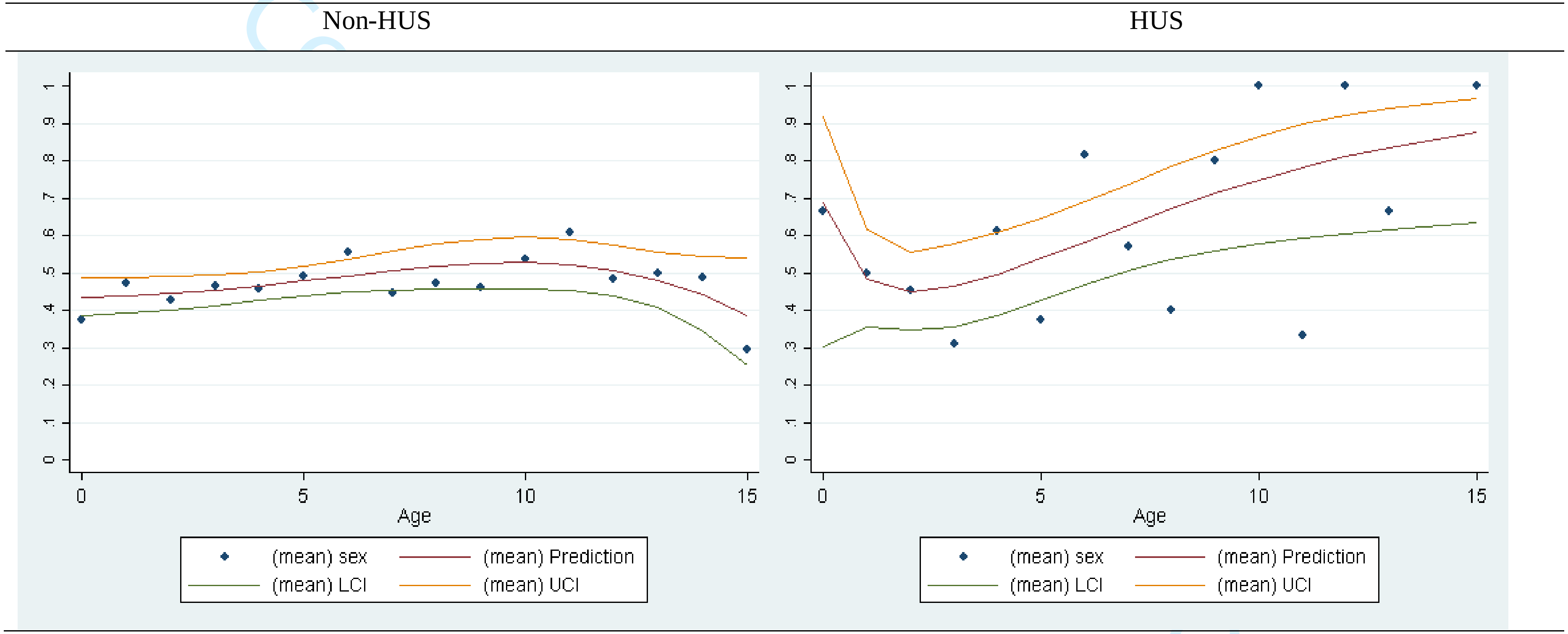

9

STROBE Statement—Checklist of items that should be included in reports of ***cohort studies***

	Item No	Recommendation
**Title and abstract**	1	(*a*) Indicate the study's design with a commonly used term in the title or the abstract
		(*b*) Provide in the abstract an informative and balanced summary of what was done and what was found
**Introduction**		
Background/rationale	2	Explain the scientific background and rationale for the investigation being reported
Objectives	3	State specific objectives, including any prespecified hypotheses
**Methods**		
Study design	4	Present key elements of study design early in the paper
Setting	5	Describe the setting, locations, and relevant dates, including periods of recruitment, exposure, follow-up, and data collection
Participants	6	(*a*) Give the eligibility criteria, and the sources and methods of selection of participants. Describe methods of follow-up
		(*b*) For matched studies, give matching criteria and number of exposed and unexposed
Variables	7	Clearly define all outcomes, exposures, predictors, potential confounders, and effect modifiers. Give diagnostic criteria, if applicable
Data sources/ measurement	8*	For each variable of interest, give sources of data and details of methods of assessment (measurement). Describe comparability of assessment methods if there is more than one group
Bias	9	Describe any efforts to address potential sources of bias
Study size	10	Explain how the study size was arrived at
Quantitative variables	11	Explain how quantitative variables were handled in the analyses. If applicable, describe which groupings were chosen and why
Statistical methods	12	(*a*) Describe all statistical methods, including those used to control for confounding
		(*b*) Describe any methods used to examine subgroups and interactions
		(*c*) Explain how missing data were addressed
		(*d*) If applicable, explain how loss to follow-up was addressed
		(*e*) Describe any sensitivity analyses
**Results**		
Participants	13*	(a) Report numbers of individuals at each stage of study—eg numbers potentially eligible, examined for eligibility, confirmed eligible, included in the study, completing follow-up, and analysed
		(b) Give reasons for non-participation at each stage
		(c) Consider use of a flow diagram
Descriptive data	14*	(a) Give characteristics of study participants (eg demographic, clinical, social) and information on exposures and potential confounders
		(b) Indicate number of participants with missing data for each variable of interest
		(c) Summarise follow-up time (eg, average and total amount)
Outcome data	15*	Report numbers of outcome events or summary measures over time
Main results	16	(*a*) Give unadjusted estimates and, if applicable, confounder-adjusted estimates and their precision (eg, 95% confidence interval). Make clear which confounders were adjusted for and why they were included
		(*b*) Report category boundaries when continuous variables were categorized
		(*c*) If relevant, consider translating estimates of relative risk into absolute risk for a meaningful time period

https://mc.manuscriptcentral.com/bmjpo
Confidential: For Review Only

Other analyses	17	Report other analyses done—eg analyses of subgroups and interactions, and sensitivity analyses
**Discussion**		
Key results	18	Summarise key results with reference to study objectives
Limitations	19	Discuss limitations of the study, taking into account sources of potential bias or imprecision. Discuss both direction and magnitude of any potential bias
Interpretation	20	Give a cautious overall interpretation of results considering objectives, limitations, multiplicity of analyses, results from similar studies, and other relevant evidence
Generalisability	21	Discuss the generalisability (external validity) of the study results
**Other information**		
Funding	22	Give the source of funding and the role of the funders for the present study and, if applicable, for the original study on which the present article is based

*Give information separately for exposed and unexposed groups.

Note: An Explanation and Elaboration article discusses each checklist item and gives methodological background and published examples of transparent reporting. The STROBE checklist is best used in conjunction with this article (freely available on the Web sites of PLoS Medicine at http://www.plosmedicine.org/, Annals of Internal Medicine at http://www.annals.org/, and Epidemiology at http://www.epidem.com/). Information on the STROBE Initiative is available at http://www.strobe-statement.org.

Confidential: For Review Only

BMJ Paediatrics Open

Sociodemographic and clinical risk-factors for paediatric typical haemolytic uraemic syndrome

Journal:	*BMJ Paediatrics Open*
Manuscript ID	bmjpo-2019-000465.R1
Article Type:	Original article
Date Submitted by the Author:	13-Jun-2019
Complete List of Authors:	Adams, Natalie; NIHR Health Protection Research Unit in Gastrointestinal Infections; Public Health England, National Infection Service<br>Byrne, Lisa; Public Health England, National Infection Service<br>Rose, Tanith; NIHR Health Protection Research Unit in Gastrointestinal Infections; University of Liverpool, Department of Public Health and Policy<br>Adak, Bob; NIHR Health Protection Research Unit in Gastrointestinal Infections<br>Jenkins, Claire; Public Health England, National Infection Service; NIHR Health Protection Research Unit in Gastrointestinal Infections<br>Charlett, Andre; Public Health England, National Infection Service<br>Violato, Mara; NIHR Health Protection Research Unit in Gastrointestinal Infections; University of Oxford, Health Economics Research Centre<br>O'Brien, Sarah; NIHR Health Protection Research Unit in Gastrointestinal Infections; University of Liverpool, Department of Public Health and Policy<br>Whitehead, Margaret; NIHR Health Protection Research Unit in Gastrointestinal Infections; University of Liverpool, Department of Public Health and Policy<br>Barr, Benjamin; NIHR Health Protection Research Unit in Gastrointestinal Infections; University of Liverpool, Department of Public Health and Policy<br>Taylor-Robinson, David; NIHR Health Protection Research Unit in Gastrointestinal Infections; University of Liverpool, Department of Public Health and Policy<br>Hawker, Jeremy; NIHR Health Protection Research Unit in Gastrointestinal Infections; Public Health England, National Infection Service
Keywords:	Epidemiology, Infectious Diseases

SCHOLARONE™
Manuscripts

Title Page

Title: Sociodemographic and clinical risk-factors for paediatric typical haemolytic uraemic syndrome: a retrospective cohort study

Authors:

Natalie L. Adams*[1,2,3,], Lisa Byrne[1,3], Tanith C. Rose[1,2], Goutam K. Adak[1], Claire Jenkins[1,3], Andre Charlett[3], Mara Violato[1,4], Sarah J. O'Brien[1,2], Margaret Whitehead[1,2], Benjamin Barr[1,2], David Taylor-Robinson[1,2±] and Jeremy Hawker[1,3±]

*Corresponding author

± Joint senior authors

Affiliations:

[1]NIHR Health Protection Research Unit in Gastrointestinal Infections, UK

[2]Department of Public Health and Policy, University of Liverpool, UK

[3]National Infection Service, Public Health England, UK

[4]Health Economics Research Centre, University of Oxford, Oxford, UK

Disclaimers:

The research was funded by the National Institute for Health Research Health Protection Research Unit (NIHR HPRU) in Gastrointestinal Infections at University of Liverpool in partnership with Public Health England (PHE), in collaboration with University of East Anglia, University of Oxford and the Quadram Institute. Natalie Adams is based at the University of Liverpool and Public Health England. The views expressed are those of the

Confidential: For Review Only

author(s) and not necessarily those of the NHS, the NIHR, the Department of Health or Public Health England.

Running title:

Paediatric HUS in England 2011-2014

Corresponding author contact information:

Dr Natalie L Adams, Gastrointestinal Infections Department, National Infection Service, Public Health England, 61 Colindale Ave, Colindale NW9 5EQ, UK

Natalie.Adams@phe.gov.uk

Word count (abstract): 243/250

Word count (text): 2727/2500

References: 32/40

Confidential: For Review Only

ABSTRACT

Objectives

Haemolytic Uraemic Syndrome (HUS) following Shiga toxin-producing *Escherichia coli* (STEC) infection is the commonest cause of acute renal failure among children in the United Kingdom. This study explored differential progression from STEC to HUS by social, demographic and clinical risk factors.

Methods

We undertook a retrospective cohort study linking two datasets. We extracted data on paediatric STEC and HUS cases identified in the Public Health England National Enhanced Surveillance System for STEC and British Paediatric Surveillance Unit HUS surveillance from October 1 2011 to October 31 2014. Using logistic regression, we estimated the odds of HUS progression by risk factors.

Results

1059 paediatric STEC cases were included in the study, of which 207 (19.55%, 95% CI 17-22%) developed HUS. In the fully-adjusted model, the odds of progression to HUS were highest in those aged 1-4 (OR 4.93, 95% CI 2.30-10.56, compared to 10-15 years), were infected with an stx2-only strain (OR 5.92, 95%CI 2.49-14.10), were prescribed antibiotics (OR 8.46, 95% CI 4.71-15.18), had bloody diarrhoea (OR 3.56, 95%CI 2.04-6.24) or vomiting (OR 4.47, 95%CI 2.62-7.63), but there was no association with progression to HUS by socioeconomic circumstances or rurality.

Conclusion

Confidential: For Review Only

Combining data from an active clinical surveillance system for HUS with the national enhanced STEC surveillance system suggests that 20% of diagnosed paediatric STEC infections in England resulted in HUS No relationship was found with socioeconomic status or rurality of cases, but differences were demonstrated by age, stx type and presenting symptoms.

Keywords: Health inequalities; Shiga-toxin producing *Escherichia* coli; STEC; Haemolytic Uraemic Syndrome; HUS

Confidential: For Review Only

Confidential: For Review Only

INTRODUCTION

Haemolytic uraemic syndrome (HUS) is a rare but serious complication of infection with Shiga toxin-producing *Escherichia coli* (STEC), affecting the blood, kidneys and, in the most severe cases, the central nervous system. Children and the elderly are considered to be the most susceptible age groups and HUS is recognised as the most common cause of acute renal failure among children in the United Kingdom (1). Strains of STEC encoding s*tx2* toxin genes are more often associated with HUS than other strains (1-6). STEC serogroup O157 is the most frequently reported strain causing illness in England. Transmission to humans occurs through consumption of contaminated food or water, exposure to a contaminated environment involving direct or indirect contact with animals or their faeces and person-to-person spread.

It is estimated that progression to HUS following STEC infection could be as high as 15% in young children (2, 7). Several studies have suggested that development of HUS varies by some demographic characteristics; higher incidence of HUS has been documented in children (particularly aged 1-4), females (particularly aged over 10 years) and in those of White ethnicity (2, 6, 8-13); however few have documented progression to HUS by other demographic characteristics such as deprivation, foreign travel, rurality or region. There is evidence to suggest that those who are disadvantaged have a lower risk of STEC infection (14-16), and potentially a lower risk of progression to HUS outside of England (16,17), however no studies have looked at the relationship between SES, STEC and HUS in England. This study aims to investigate the relationship between demographic factors, STEC infection and subsequent development of HUS in a well-characterised paediatric population in England with high case-ascertainment.

METHODS

Data, setting and source

We undertook a retrospective cohort study linking two data sources; the Public Health England (PHE) National Enhanced Surveillance System for STEC (NESSS) and the British Paediatric Surveillance Unit (BPSU) HUS Study in conjunction with PHE. The linkage of two robust datasets, both of which can record HUS status, ensures high ascertainment of HUS cases. Firstly, we extracted data on STEC cases aged 0-15 years (inclusive) identified in NESSS during the period of the BPSU HUS Study (October 1 2011 to October 31 2014). All laboratory-confirmed STEC cases in England are reported by NHS laboratories to Public Health England staff who collect standardised data through an enhanced surveillance questionnaire (ESQ) as part of their public health response: this standardised dataset is collated centrally in NESSS for further validation and analysis. The ESQ collects detailed information on patient demographics, symptoms, food and water exposures and UK and non-UK travel during the exposure period (the week prior to illness onset). When a presumptive STEC is identified at the local laboratory or a case of HUS is identified, specimens are sent to PHE Gastrointestinal Bacteria Reference Unit (GBRU) for testing and patient ESQs are linked to microbiological results. Due to the timing of the ESQ administration in NESSS (which is designed to inform the acute public health response), this system can under-ascertain HUS as this can develop after completion of the questionnaire. This surveillance system is described in detail elsewhere (2).

Secondly, we extracted clinical data on paediatric (<16 years) HUS cases, collected by the BPSU HUS Study, an active surveillance system requiring regular returns from clinicians. Within this study, data were captured using a standardised questionnaire administered to paediatricians collecting information on; case demography; treatment history; microbiological investigations; clinical parameters of illness; clinical management of illness and status of the case at the time of data capture. Cases in the BPSU dataset were linked on

https://mc.manuscriptcentral.com/bmjpo

Confidential: For Review Only

National Health Service (NHS) number, which was available for all cases, to those in the NESSS dataset to create a retrospective cohort. Supplementary Figure 1 provides details of the selection of study participants.

For statistical analysis, cases for whom no microbiological information was available (n=4) and cases identified via serological testing only (n=66) were excluded in order to assess the role of *stx* subtype. Due to missing data for the ethnicity variable (19.1%), we used multiple imputation using chained equations to impute values where ethnicity (white/non-white) was missing for statistical analysis. Fifty imputed datasets were generated. The distribution of ethnicity by age, sex and region was assessed to check the missing at random (MAR) assumption. There was no difference in missing ethnicity by sex, however there were some differences by age group (57.3% of cases with missing ethnicity were in the 1-4 age group; n=114/199) and region (31.2% of cases with missing ethnicity were in London; n=62/199); these were not regarded as problematic however as, given the observed data for other variables, the missing data were considered independent.

Ethics

Ethical approval was originally obtained for the main study (Ref: 11/LO/1412). As of October 2010, HUS is a statutory reportable condition and this study falls under the existing Health Protection Agency (now Public Health England) permissions under Section 251 of the NHS Act 2006. In addition, we received a favourable ethical opinion from the South East Coast - Surrey Research Ethics Committee (15/LO/2138) on 1 December 2015 covering the use of this dataset for this study.

Patient involvement

Patients were not directly involved in the design of this study.

Confidential: For Review Only

Outcome and exposures

The outcome of interest was HUS, determined by the case meeting the BPSU clinical criteria (See supplementary Table 1) (18) or completion of the HUS field in the ESQ. Covariates in the analysis were age group (<1, 1-4, 5-9, 10-15 years); sex (male/female); ethnicity (White/non-White); travel (yes/no); rurality (rural/urban); microbiology (Shiga-toxin (*Stx*)); antibiotic use (yes/no); clinical symptoms (diarrhoea, bloody diarrhoea, nausea, vomiting, abdominal pain, fever) and region of residence. The s*tx* type, the primary STEC virulence factor, was used as the main microbiological variable (19). Where symptoms, travel status and healthcare contact variables were blank or unknown, these were recoded as a negative response. As a proxy for childhood socioeconomic circumstances (SECs) we used a small-area deprivation measure, the Index of Multiple Deprivation 2010 (IMD) (20), assigned to each case based on their postcode and divided into population-level quintiles, with the first quintile representing the least deprived and the fifth quintile representing the most deprived.

Analysis strategy

Comparisons of proportions were tested using the chi-squared test. We explored univariate relationships between progression to HUS and the covariates of interest before fitting a multivariate logistic regression model. All variables were retained in this model in order to control for any potential confounding. Interaction terms between variables (IMD, ethnicity, age and sex) were tested to investigate whether the strength of any relationship was moderated by the inclusion of another variable. Analyses were conducted in Stata 13.1 (Statacorp, Texas).

Robustness tests

Confidential: For Review Only

We performed multiple sensitivity analyses to test the validity of the main analysis; first by (i) excluding cases that were likely to have a travel-acquired STEC infection (date of onset is within one exposure period, 7 days, of having returned from outside of the UK) and (ii) separately excluding cases with unknown ethnicity to determine whether there were differences in progression to HUS by SECs for children who travelled abroad during their incubation period compared to those who did not or those with ethnicity recorded and those without respectively.

RESULTS

Descriptive analysis

Of 1059 paediatric STEC cases included in the study, 207 (19.55%, 95% CI 17.27%-22.04%) developed HUS. Progression to HUS varied by age and gender (Table 1), the highest was observed in females aged 1-4 years (26.0%). A higher proportion of progression to HUS was observed in females aged 10-15 years compared to males of the same age (19.3%, 95% CI 12.3-27.9 versus 7.1%, 95% CI 2.9-14.2, p=0.01), and amongst females aged less than 1 year compared to males of the same age, although this was not significant (14.3%, 95% CI 4.0-32.7 versus 4.8%, 95% CI 0.6-16.2, p=0.16). Although progression to HUS was higher in the least disadvantaged quintile (47/245, 19.2%, 95% CI 14.4-24.7%) compared with the most disadvantaged quintile (29/189, 15.3%, 95% CI 10.5-21.3%) this difference was not statistically significant (p=0.29). The highest proportion progressing to HUS was in quintile 3 (53/219, 24.2%, 95% CI 18.7-30.4%) and there was no clear pattern across the 5 quintiles (p=0.07; quintile 2 - 35/221, 15.8%, 95% CI 11.3-21.3%; quintile 4 - 43/185, 23.2%, 95% CI 17.4-30%).

Multivariable analysis

Confidential: For Review Only

In the fully adjusted model (Table 2), there were significantly lower odds of HUS amongst <1, 5-9 and 10-15 year olds compared to 1-4 year olds and significantly higher odds of HUS amongst those infected with s*tx*2-only strains, those prescribed antibiotics and among those who had experienced bloody diarrhoea or vomiting . The most disadvantaged children had lower odds of progression to HUS compared to the least disadvantaged children (OR 0.57, 95% CI 0.25-1.31) but the difference was not significant. There was no statistically significant difference in risk by rurality (OR 0.88, 95% CI 0.52-1.48) or by region (Table 2). There were no significant interactions identified (data not shown).

The sensitivity analyses conducted to assess the robustness of the findings did not alter the overall conclusions of this research (Supplementary Tables 2 and 3).

DISCUSSION

In a novel linkage and analysis of two datasets with high case-ascertainment to explore the role of demographic and socioeconomic factors in the development of HUS following STEC infection, we found progression from STEC infection to HUS to be 20% in this paediatric cohort in England. Odds of HUS progression varied by age, stx type, antibiotic exposure and clinical presentation, with children aged 1-4 years infected with stx2-only, with reported antibiotic exposure and presenting with bloody diarrhoea or vomiting at highest risk. Few studies have explored the social patterning of risk factors for STEC (21) or the socio-demographic risk factors associated with progression to HUS, and no such studies have been undertaken in England. We found no relationship between progression to HUS and socioeconomic status in children in this study.

Our study has several strengths. This study captures the progression of HUS in a well-characterised paediatric STEC population. To the best of our knowledge, as confirmed by a prior review of the literature and discussion with national experts, this is the first study to

Confidential: For Review Only

combine a prospective active surveillance system and a multisource national surveillance system to study the risk factors for HUS and as such is likely to have better case-ascertainment of HUS than previous studies and is related to good STEC denominators. Furthermore, this study makes use of one of the largest cohorts of HUS cases. The results of this study are likely to be generalisable to other high-income countries with a similar pattern of STEC infection. Despite this, there are some limitations. It is possible that there is residual confounding that could not be controlled for, such as intrinsic childhood characteristics which may increase differential vulnerability or susceptibility by SEC such as genetic predisposition, co-morbidities, and clinical or treatment characteristics. Further, as an area-level measure of SEC was used, it is possible that it may not have been sensitive enough to detect the effect of socioeconomic inequalities, particularly if individual factors rather than area-level factors have more influence over the risk of acquiring more severe strains of STEC with increased risk of progression to HUS. However, person-to-person spread is an important risk factor for GI infections and, although there is a risk of ecological fallacy, area-level measures have the advantage of including potential environmental factors such as housing and living environment deprivation which are likely to be important factors in considering individual risk of infection. Excluding individuals with a serological result only from the statistical analysis may introduce a potential bias leading to an underestimate of HUS incidence, which may be important if there are geographical or host-factors which are linked to severity of illness, although the number of serology-only diagnoses was small. In England, most diagnosed cases of STEC are of serogroup O157 (95% in our study), and it is possible that our results may be biased towards the relationship between STEC O157 and progression to HUS, which may differ if other, possibly less pathogenic, serogroups predominate. It is possible however, that the risk of progression to HUS could be different in populations exposed to STEC organisms with a lower proportion of stx2-only producing strains, or with a

Confidential: For Review Only

different age distribution of cases. There were also some missing data in our study, particularly for ethnicity, which we addressed using multiple imputation. The ethnicity variable used (White/Non-White) was also crude and adopted because of data quality issues. This may mask differences in socioeconomic status. No data were available on whether the children included in our study had underlying or chronic conditions which may be related to their risk of developing HUS. Finally, it was not always possible to determine whether antibiotics had been prescribed during treatment for STEC infection or following a diagnosis of HUS therefore the relevant association should be interpreted with caution.

The finding of 19.5% (95% CI 17-22%) of diagnosed STEC cases progressing to HUS is higher than previous studies, which have estimated the proportion of paediatric cases of STEC O157 progressing to HUS to be 15% (95% CI 11-19%) in females aged 1-4 years in England (2) and 15.3% (95% CI 13-18%) in children aged <5 years in the USA (7). Our study uses data derived from two linked surveillance systems providing high ascertainment of both STEC and HUS cases which provides a more robust estimate. It is likely that there will also be a bias resulting from ascertainment of STEC cases from laboratory specimens, as milder cases of gastrointestinal infection are less likely to be microbiologically tested, but this will also be true of previous published studies.

Whilst rurality has been reported as an important factor in risk for STEC infection (2, 14), our study suggests that rurality is not a significant driver of progression to HUS. It is important to note that there are environmental factors, such as cattle density, that were not included in this study and which may be more important factors in risk of STEC infection. Our finding that rurality was not linked to progression to HUS following STEC infection may also be due to the majority of our cases (95%) being STEC O157 – this finding may be different in more heterogenous dataset from countries with greater variability by serogroup. Similarly, despite evidence to suggest that the risk and consequences of GI infections in general are greater for

Confidential: For Review Only

disadvantaged children (22-26) – the finding in our study suggests that lower childhood SEC is unlikely to be a contributor for development of HUS.

Previous studies in England have suggested that children aged 1-4 years, females and white ethnic groups have the highest incidence of STEC infection (2, 27). Our study echoes the findings by Milford et al (6) which demonstrated higher progression to HUS amongst children aged 1-4 years. No overall difference in risk of HUS by sex was identified in our study, a finding echoed in several other previous studies (28-31); this is an area of disagreement in the literature with several studies finding higher risk amongst women (7, 17, 32) although two of these studies finding higher risk in women did not look specifically among children (7, 32). We did find differences in risk by sex within specific age groups, with a greater proportion of progression to HUS amongst girls less than 1 year of age and 10-15 years of age compared to boys of the same age groups (Table 1), although no significant interaction between age and sex could be identified. The reasons for the differential risk by age are currently unclear and call for a deeper understanding of differences in risks and exposures between these groups.

The association between clinical presentation with vomiting and bloody diarrhoea and increased risk of HUS reported in this study has been identified previously (12) and, as such, the presence of these symptoms particularly in paediatric STEC cases should evoke a high level of clinical suspicion for the potential development of HUS.

Our study quantifies the proportion of paediatric STEC cases progressing to HUS in a well – defined population with high ascertainment. It also quantifies the risk factors associated with progression to HUS in terms of sociodemographic characteristics as well as clinical presentation. Further research is warranted to elucidate the populations at risk of STEC

https://mc.manuscriptcentral.com/bmjpo

Confidential: For Review Only

infection and HUS in terms of deprivation, ethnicity, age and sex, in order to better understand whether there are real differences in risk or artefacts of surveillance.

FUNDING

The research was funded by the National Institute for Health Research Health Protection Research Unit (NIHR HPRU) in Gastrointestinal Infections [grant number NIHR HPRU 201210,038] at University of Liverpool in partnership with Public Health England (PHE), in collaboration with University of East Anglia, University of Oxford and the Quadram Institute. Natalie Adams is based at the University of Liverpool and Public Health England. The views expressed are those of the author(s) and not necessarily those of the NHS, the NIHR, the Department of Health and Social Care or Public Health England. David Taylor-Robinson is funded by the MRC on a Clinician Scientist Fellowship (MR/P008577/1). Ben Barr and Tanith Rose were also supported by the National Institute for Health Research (NIHR) Collaboration for Leadership in Health Research and Care (CLAHRC NWC).

CONFLICT OF INTEREST

All authors: No potential conflicts of interest.

ACKNOWLEDGMENTS

This work was funded by the National Institute for Health Research Health Protection Research Unit in Gastrointestinal Infections. The authors would like to thank Kirsten Glen and Naomi Launders for their work on the National Enhanced Surveillance System for STEC, Ross Harris for statistical advice and Richard Lynn at the British Paediatric Surveillance Unit for facilitating the BPSU HUS study.

CONFLICT OF INTEREST

Confidential: For Review Only

The authors declare that they have no conflicts of interest.

Confidential: For Review Only

WHAT IS ALREADY KNOWN ON THIS TOPIC

- HUS is recognised as the most common cause of acute renal failure among children in the United Kingdom.
- It is estimated that progression to HUS following STEC infection could be as high as 15% in young children.
- Several studies have suggested that development of HUS varies by demographic characteristics however few have documented progression to HUS by demographic characteristics.

WHAT THIS STUDY ADDS

- A fifth of paediatric STEC cases developed the serious complication of haemolytic uraemic syndrome in England.
- This figure is higher than previously reported in England, and varied by demographic and clinical factors
- Socioeconomic factors did not influence progression to SES.

Confidential: For Review Only

REFERENCES

1. Lynn RM, O'Brien SJ, Taylor CM, et al. Childhood hemolytic uremic syndrome, United Kingdom and Ireland. Emerging Infectious Diseases. 2005;11(4):590-6. doi:10.3201/eid1104.040833
2. Byrne L, Jenkins C, Launders N, Elson R, Adak GK. The epidemiology, microbiology and clinical impact of Shiga toxin-producing Escherichia coli in England, 2009-2012. Epidemiology and Infection. 2015:1-13. doi:10.1017/S0950268815000746
3. Ethelberg S, Olsen KE, Scheutz F, et al. Virulence factors for hemolytic uremic syndrome, Denmark. Emerging Infectious Diseases. 2004;10(5):842-7. doi:10.3201/eid1005.030576
4. Persson S, Olsen KE, Ethelberg S, Scheutz F. Subtyping method for Escherichia coli shiga toxin (verocytotoxin) 2 variants and correlations to clinical manifestations. J Clin Microbiol. 2007;45(6):2020-4. doi:10.1128/JCM.02591-06
5. Dallman TJ, Ashton PM, Byrne L, et al. Applying phylogenomics to understand the emergence of Shiga-toxin-producing Escherichia coli O157:H7 strains causing severe human disease in the UK. Microbial Genomics. 2015;1(3). doi:doi:10.1099/mgen.0.000029
6. Milford DV, Taylor CM, Guttridge B, Hall SM, Rowe B, Kleanthous H. Haemolytic uraemic syndromes in the British Isles 1985-8: association with verocytotoxin producing Escherichia coli. Part 1: Clinical and epidemiological aspects. Archives of Disease in Childhood. 1990;65(7):716-21.
7. Gould LH, Demma L, Jones TF, et al. Hemolytic uremic syndrome and death in persons with Escherichia coli O157:H7 infection, foodborne diseases active surveillance network sites, 2000-2006. Clinical Infectious Diseases. 2009;49(10):1480-5. doi:10.1086/644621
8. Kinney JS, Gross TP, Porter CC, Rogers MF, Schonberger LB, Hurwitz ES. Hemolytic-uremic syndrome: a population-based study in Washington, DC and Baltimore, Maryland. American Journal of Public Health. 1988;78(1):64-5.
9. Rogers MF, Rutherford GW, Alexander SR, et al. A population-based study of hemolytic-uremic syndrome in Oregon, 1979-1982. American Journal of Epidemiology. 1986;123(1):137-42.
10. Tarr PI, Hickman RO. Hemolytic uremic syndrome epidemiology: a population-based study in King County, Washington, 1971 to 1980. Pediatrics. 1987;80(1):41-5.
11. Bell WR. Thrombotic thrombocytopenic purpura/hemolytic uremic syndrome relapse: frequency, pathogenesis, and meaning. Seminars in hematology. 1997;34(2):134-9.
12. Launders N, Byrne L, Jenkins C, Harker K, Charlett A, Adak GK. Disease severity of Shiga toxin-producing E. coli O157 and factors influencing the development of typical haemolytic uraemic syndrome: a retrospective cohort study, 2009–2012. BMJ open. 2016;6(1). doi:10.1136/bmjopen-2015-009933
13. Elliott EJ, Robins-Browne RM, O'Loughlin EV, et al. Nationwide study of haemolytic uraemic syndrome: clinical, microbiological, and epidemiological features. Arch Dis Child. 2001;85:125-31.
14. Chang M, Groseclose SL, Zaidi AA, Braden CR. An ecological analysis of sociodemographic factors associated with the incidence of salmonellosis, shigellosis, and E. coli O157:H7 infections in US counties. Epidemiology and infection. 2009;137(6):810-20. doi:10.1017/s0950268808001477
15. Jalava K, Ollgren J, Eklund M, Siitonen A, Kuusi M. Agricultural, socioeconomic and environmental variables as risks for human verotoxigenic Escherichia coli (VTEC) infection in Finland. BMC Infect Dis. 2011;11:275. doi: http://dx.doi.org/10.1186/1471-2334-11-275. PubMed PMID: 22008456; PubMed Central PMCID: PMCPMC3226588.

Confidential: For Review Only

16. Whitney BM, Mainero C, Humes E, Hurd S, Niccolai L, Hadler JL. Socioeconomic status and foodborne pathogens in Connecticut, USA, 2000–20111. Emerging Infectious Diseases. 2015;21(9):1617-24. doi: 10.3201/eid2109.150277.
17. Rowe PC, Orrbine E, Wells GA, McLaine PN. Epidemiology of hemolytic-uremic syndrome in Canadian children from 1986 to 1988. The Canadian Pediatric Kidney Disease Reference Centre. Journal of Pediatrics. 1991;119(2):218-24.
18. Byrne L, on behalf of the BPSU HUS Study Team,. BPSU HUS Study Report. 2017.
19. Ethelberg S, Olsen KE, Scheutz F, et al. Virulence factors for hemolytic uremic syndrome, Denmark. Emerg Infect Dis. 2004;10(5):842-7. doi:10.3201/eid1005.030576
20. Department for Communities and Local Government. English Indices of Deprivation 2010. Department for Communities and Local Government. 2011. https://www.gov.uk/government/uploads/system/uploads/attachment_data/file/6871/1871208.pdf. Accessed 16/09/2016.
21. Bentancor AB, Ameal LA, Calvino MF, Martinez MC, Miccio L, Degregorio OJ. Risk factors for Shiga toxin-producing Escherichia coli infections in preadolescent schoolchildren in Buenos Aires, Argentina. Journal of Infection in Developing Countries. 2012;6(5):378-86.
22. Olowokure B, Hawker J, Weinberg J, Gill N, Sufi F. Deprivation and hospital admission for infectious intestinal diseases. Lancet. 1999;353(9155):807-8. doi:10.1016/S0140-6736(99)00611-X
23. Phillips G, Tam CC, Rodrigues LC, Lopman B. Risk factors for symptomatic and asymptomatic norovirus infection in the community. Epidemiology and Infection. 2011;139(11):1676-86. doi:http://dx.doi.org/10.1017/S0950268810002839
24. Pockett RD, Adlard N, Carroll S, Rajoriya F. Paediatric hospital admissions for rotavirus gastroenteritis and infectious gastroenteritis of all causes in England: an analysis of correlation with deprivation. Current medical research and opinion. 2011;27(4):777-84. doi:10.1185/03007995.2011.555757
25. Rose TC, Adams NL, Barr B, et al. Socioeconomic status is associated with symptom severity and sickness absence in people with infectious intestinal disease in the UK. BMC Infect Dis. 2017;17(1):447. doi:10.1186/s12879-017-2551-1
26. Adams NL, Rose TC, Hawker J, et al. Relationship between socioeconomic status and gastrointestinal infections in developed countries: A systematic review and meta-analysis. PloS one. 2018;13(1):e0191633. doi:10.1371/journal.pone.0191633
27. Adams NL, Byrne L, Smith GA, et al. Shiga Toxin-Producing Escherichia coli O157, England and Wales, 1983-2012. Emerging Infectious Diseases. 2016;22(4):590-7. doi:10.3201/eid2204.151485
28. Tserenpuntsag B, Chang H-G, Smith PF, Morse DL. Hemolytic uremic syndrome risk and Escherichia coli O157:H7. Emerging Infectious Diseases. 2005;11(12):1955-7.
279 Bell BP, Griffin PM, Lozano P, Christie DL, Kobayashi JM, Tarr PI. Predictors of hemolytic uremic syndrome in children during a large outbreak of Escherichia coli O157:H7 infections. Pediatrics. 1997;100(1):E12.
30. Rowe PC, Orrbine E, Lior H, et al. Risk of hemolytic uremic syndrome after sporadic Escherichia coli O157:H7 infection: results of a Canadian collaborative study. Investigators of the Canadian Pediatric Kidney Disease Research Center. Journal of Pediatrics. 1998;132(5):777-82.
31. Cimolai N, Basalyga S, Mah DG, Morrison BJ, Carter JE. A continuing assessment of risk factors for the development of Escherichia coli O157:H7-associated hemolytic uremic syndrome. Clinical Nephrology. 1994;42(2):85-9.
32. Chang H-GH, Tserenpuntsag B, Kacica M, Smith PF, Morse DL. Hemolytic uremic syndrome incidence in New York. Emerging Infectious Diseases. 2004;10(5):928-31.

https://mc.manuscriptcentral.com/bmjpo

TABLES

Table 1: Characteristics of cohort participants by HUS status (n=1,059)

		No HUS n (%)	HUS n (%)
**Total**		852 (80.5)	207 (19.6)
**Age group**	<1	64 (91.4)	6 (8.6)
	1-4	370 (76.1)	116 (23.9)
	5-9	239 (80.7)	57 (19.3)
	10-15	179 (86.5)	28 (13.5)
**Sex**	Female	400 (77.5)	116 (22.5)
	Male	452 (83.2)	91 (16.8)
**Age and Sex**	Female <1	24 (85.7)	4 (14.3)
	Female 1-4	171 (74.0)	60 (26.0)
	Female 5-9	117 (79.1)	31 (20.9)
	Female 10-15	88 (80.7)	21 (19.3)
	Male <1	40 (95.2)	2 (4.8)
	Male 1-4	199 (78.0)	56 (22.0)
	Male 5-9	122 (82.4)	26 (17.6)
	Male 10-15	91 (92.9)	7 (7.1)
**Ethnicity**	White	552 (80.5)	134 (19.5)
	Non-white	138 (88.5)	18 (11.5)
	Unknown	162 (74.7)	55 (23.4)
**IMD Quintile**	1 (Least Disadvantaged)	198 (80.8)	47 (19.2)
	2	186 (84.2)	35 (15.8)
	3	166 (75.8)	53 (24.2)
	4	142 (76.8)	43 (23.2)
	5 (Most Disadvantaged)	160 (84.7)	29 (15.3)
**Travel**	Yes	128 (85.3)	22 (14.7)
	No	724 (79.7)	185 (20.4)
**Rurality**	Rural	230 (80.4)	56 (19.6)
	Urban	622 (80.5)	151 (19.5)
**Region**	East Midlands	65 (81.3)	15 (18.8)
	East of England	57 (80.3)	14 (19.7)
	London	93 (81.6)	21 (18.4)
	North East	64 (77.1)	19 (22.9)
	North West	153 (77.7)	44 (22.3)
	South East	92 (78.6)	25 (21.4)
	South West	101 (75.9)	32 (24.1)
	West Midlands	96 (84.2)	18 (15.8)
	Yorkshire and Humber	131 (87.3)	19 (12.7)
Stx	*Stx*1	17 (94.4)	1 (5.6)
	*Stx*2	609 (81.7)	136 (18.3)
	*Stx*1+2	219 (96.9)	7 (3.1)
	Serology	7 (10.6)	59 (89.4)
	Unknown	0 (0.0)	4 (100.0)
**Symptoms**	Diarrhoea	803 (80.3)	197 (19.7)
	Bloody diarrhoea	432 (74.0)	152 (26.0)
	Nausea	278 (75.8)	89 (24.3)
	Vomiting	330 (66.1)	169 (33.9)
	Abdominal pain	574 (78.2)	160 (21.8)

Confidential: For Review Only

	Fever	273 (76.7)	83 (23.3)
**Healthcare contact**	Antibiotics	53 (40.8)	77 (59.2)
	NHS Direct	67 (72.0)	26 (28.0)
	GP	570 (83.7)	111 (16.3)
	A&E	186 (66.9)	92 (33.1)
	Other healthcare contact	98 (74.8)	33 (25.2)
	Hospital	223 (52.4)	203 (47.6)

HUS – haemolytic uraemic syndrome; *stx* – Shiga toxin; NHS Direct – National Health Service telephone advice line, now NHS 111; GP – General Practitioner; A&E – accident and emergency

Confidential: For Review Only

Table 2: Adjusted and unadjusted regression analysis (n=989)

Variable	Category	n (%)	Unadjusted		Adjusted[a]		p value[b]
			Odds Ratio	(95% CI)	Odds Ratio	(95% CI)	
**Age group**	<1	67 (6.8)	0.19	(0.06-0.62)	**0.21**	**(0.05-0.82)**	**0.03**
	1-4	456 (46.1)	1.0 (reference)		1.0 (reference)		
	5-9	274 (27.7)	0.62	(0.40-0.94)	**0.43**	**(0.25-0.74)**	**0.002**
	10-15	192 (19.4)	0.34	(0.19-0.61)	**0.20**	**(0.09-0.43)**	**<0.001**
**Sex**	Male	513 (51.9)	1.0 (reference)		1.0 (reference)		
	Female	476 (48.1)	1.37	(0.96-1.96)	1.38	(0.88-2.14)	0.16
**Ethnicity[±]**	White	797 (80.6)	1.0 (reference)		1.0 (reference)		
	Non-White	192 (19.4)	0.39	(0.18-0.81)	**0.28**	**(0.11-0.74)**	**0.01**
**Travel**	No	850 (86.0)	1.0 (reference)		1.0 (reference)		
	Yes	139 (14.0)	0.46	(0.24-0.88)	0.64	(0.28-1.45)	0.28
**Rurality**	Urban	719 (72.7)	1.0 (reference)		1.0 (reference)		
	Rural	270 (27.3)	1.21	(0.82-1.77)	0.88	(0.52-1.48)	0.63
**IMD Quintile**	1 (least disadvantaged)	231 (23.4)	1.0 (reference)		1.0 (reference)		
	2	210 (21.2)	0.83	(0.48-1.42)	0.64	(0.32-1.27)	0.20
	3	204 (20.6)	1.28	(0.77-2.12)	1.01	(0.54-1.91)	0.97
	4	170 (17.2)	1.10	(0.64-1.90)	1.10	(0.54-2.26)	0.79
	5 (most disadvantaged)	174 (17.6)	0.57	(0.30-1.06)	0.57	(0.25-1.31)	0.18
**Region**	East Midlands	72 (7.3)	0.62	(0.24-1.59)	0.59	(0.18-1.92)	0.39
	East of England	66 (6.7)	1.03	(0.44-2.42)	1.12	(0.37-3.37)	0.84
	London	108 (10.9)	1.0 (reference)		1.0 (reference)		
	North East	76 (7.7)	1.19	(0.53-2.64)	0.71	(0.26-1.97)	0.51
	North West	185 (18.7)	1.20	(0.63-2.31)	1.02	(0.44-2.37)	0.97
	South East	107 (10.8)	1.09	(0.52-2.28)	1.31	(0.48-3.63)	0.60
	South West	127 (12.8)	1.48	(0.75-2.93)	1.25	(0.50-3.13)	0.63
	West Midlands	104 (10.5)	0.54	(0.23-1.29)	0.53	(0.18-1.53)	0.24
	Yorkshire and Humber	144 (14.6)	0.62	(0.29-1.33)	0.52	(0.20-1.34)	0.17
Stx	*Stx*1+2	226 (22.9)	1.0 (reference)		1.0 (reference)		
	*Stx*1	18 (1.8)	1.84	(0.21-15.84)	5.53	(0.53-57.42)	0.15
	*Stx*2	745 (75.3)	6.99	(3.22-15.17)	**5.92**	**(2.49-14.10)**	**<0.001**
**Antibiotics**	No	887 (89.7)	1.0 (reference)		1.0 (reference)		

Confidential: For Review Only

	Yes	102 (10.3)	8.54	(5.48-13.30)	**8.46**	**(4.71-15.18)**	**<0.001**
**Diarrhoea**	No	49 (5.0)	1.0 (reference)		1.0 (reference)		
	Yes	940 (95.0)	8.61	(1.18-62.89)	4.04	(0.50-32.59)	0.19
**Bloody diarrhoea**	No	440 (44.5)	1.0 (reference)		1.0 (reference)		
	Yes	549 (55.5)	4.85	(3.07-8.00)	**3.56**	**(2.04-6.24)**	**<0.001**
**Nausea**	No	653 (66.0)	1.0 (reference)		1.0 (reference)		
	Yes	336 (34.0)	1.52	(1.06-2.18)	1.12	(0.67-1.86)	0.66
**Vomiting**	No	549 (55.5)	1.0 (reference)		1.0 (reference)		
	Yes	440 (44.5)	6.05	(3.95-9.26)	**4.47**	**(2.62-7.63)**	**<0.001**
**Abdominal pain**	No	309 (31.2)	1.0 (reference)		1.0 (reference)		
	Yes	680 (68.8)	1.49	(0.99-2.25)	0.82	(0.46-1.46)	0.50
**Fever**	No	657 (66.4)	1.0 (reference)		1.0 (reference)		
	Yes	332 (33.6)	1.50	(1.05-2.16)	1.05	(0.67-1.66)	0.82

[a]Adjusted for all other covariates in the model; [b]Statistical significance of relationship between HUS and each variable tested using χ^2 test ; *stx* – Shiga toxin gene; [±]Multiply imputed variable

Confidential: For Review Only

SUPPLEMENTARY DATA

Supplementary Table 1a: Clinical case definition for HUS

A child (aged <16 years of age) who has:
Acute kidney injury (AKI) defined by oligoanuria and/or elevated creatinine for age<br><br>AND<br><br>Microangiopathic haemolytic anamia (MAHA) defined by haemoglobin level <10 g/L with fragmented erythrocytes<br><br>AND/OR<br><br>Thrombocytopenia defined by a platelet count of <130,000 × 109/L<br><br>WITHOUT septicaemia, malignant hypertension, chronic uraemia, or primary vascular disease<br><br>Elevated creatinine levels differed by age group and were those above the thresholds in Table 1

Supplementary Table 1b: Creatinine level (micromol/L) thresholds by age group

**Age Group**	**Normal Creatinine threshold (micromol/L)**
0-7 days	100
8-14 days	80
15-28 days	55
1m-3 years	40
4-6 years	46
7-9 years	56
10-12 years	60
13-15 years	80

Confidential: For Review Only

Supplementary Table 2: Adjusted and unadjusted regression analysis - Sensitivity analysis excluding travel cases (n subjects=850)

Variable	Category	n (%)	Unadjusted		Adjusted[a]		p value
			Odds Ratio	(95% CI)	Odds Ratio	(95% CI)	
**Age group**	<1	55 (6.5)	0.14	(0.03-0.59)	**0.12**	**(0.02-0.62)**	**0.01**
	1-4	400 (47.1)	1.0 (reference)		1.0 (reference)		
	5-9	242 (28.5)	0.61	(0.39-0.94)	**0.45**	**(0.25-0.79)**	**0.005**
	10-15	153 (18.0)	0.32	(0.17-0.60)	**0.17**	**(0.07-0.39)**	**<0.001**
**Sex**	Male	445 (52.4)	1.0 (reference)		1.0 (reference)		
	Female	405 (47.7)	1.31	(0.91-1.90)	1.30	(0.81-2.08)	0.27
**Ethnicity**	White	700 (82.4)	1.0 (reference)		1.0 (reference)		
	Non-White	150 (17.6)	0.41	(0.20-0.83)	**0.27**	**(0.10-0.73)**	**0.01**
**Rurality**	Urban	606 (71.3)	1.0 (reference)		1.0 (reference)		
	Rural	244 (28.7)	1.28	(0.86-1.90)	1.07	(0.62-1.86)	0.80
**IMD Quintile**	1 (least disadvantaged)	196 (23.1)	1.0 (reference)		1.0 (reference)		
	2	184 (21.7)	0.88	(0.50-1.54)	0.75	(0.36-1.53)	0.43
	3	180 (21.2)	1.24	(0.73-2.10)	1.06	(0.54-2.10)	0.86
	4	150 (17.7)	1.07	(0.61-1.90)	1.14	(0.53-2.46)	0.74
	5 (most disadvantaged)	140 (16.5)	0.52	(0.26-1.04)	0.57	(0.23-1.43)	0.23
**Region**	East Midlands	61 (7.2)	0.54	(0.19-1.49)	0.49	(0.14-1.80)	0.29
	East of England	55 (6.5)	0.84	(0.33-2.16)	1.02	(0.30-3.44)	0.98
	London	83 (9.8)	1.0 (reference)		1.0 (reference)		
	North East	71 (8.4)	0.90	(0.38-2.14)	0.59	(0.19-1.81)	0.35
	North West	172 (20.2)	1.13	(0.56-2.25)	1.06	(0.42-2.67)	0.90
	South East	73 (8.6)	1.27	(0.58-2.86)	1.78	(0.57-5.56)	0.32
	South West	110 (12.9)	1.38	(0.66-2.86)	1.14	(0.42-3.11)	0.80
	West Midlands	95 (11.2)	0.52	(0.21-1.26)	0.57	(0.18-1.78)	0.33
	Yorkshire and Humber	130 (15.3)	0.59	(0.27-1.32)	0.53	(0.19-1.47)	0.22
Stx	*Stx*1+2	183 (21.5)	1.0 (reference)		1.0 (reference)		
	*Stx*1	8 (0.9)	4.21	(0.45-39.89)	**24.71**	**(1.86-328.34)**	**0.02**
	*Stx*2	659 (77.5)	6.97	(3.02-16.10)	**6.08**	**(2.32-15.92)**	**<0.001**
**Antibiotics**	No	766 (90.1)	1.0 (reference)		1.0 (reference)		
	Yes	84 (9.9)	10.0	(6.18-16.32)	**10.89**	**(5.65-20.97)**	**<0.001**
**Diarrhea**	No	48 (6.6)	1.0 (reference)		1.0 (reference)		
	Yes	802 (94.4)	9.26	(1.27-67.70)	4.00	(0.48-33.16)	0.20

Confidential: For Review Only

**Bloody diarrhea**	No	360 (42.4)	1.0 (reference)		1.0 (reference)		
	Yes	490 (57.7)	5.10	(3.10-8.38)	**3.70**	**(2.00-6.85)**	**<0.001**
**Nausea**	No	562 (66.1)	1.0 (reference)		1.0 (reference)		
	Yes	288 (33.9)	1.52	(1.04-2.22)	1.03	(0.60-1.76)	0.92
**Vomiting**	No	465 (54.7)	1.0 (reference)		1.0 (reference)		
	Yes	385 (45.3)	6.50	(4.13-10.23)	**5.25**	**(2.94-9.38)**	**<0.001**
**Abdominal pain**	No	259 (30.5)	1.0 (reference)		1.0 (reference)		
	Yes	591 (69.5)	1.39	(0.91-2.13)	0.73	(0.39-1.36)	0.32
**Fever**	No	569 (66.9)	1.0 (reference)		1.0 (reference)		
	Yes	281 (33.1)	1.71	(1.17-2.50)	1.28	(0.79-2.09)	0.32

aAdjusted for all other covariates in the model; *stx* – shiga toxin gene

Confidential: For Review Only

Supplementary Table 3: Adjusted and unadjusted regression analysis - Sensitivity analysis excluding ethnicity variable (n subjects=989)

**Variable**	**Category**	**n (%)**	**Unadjusted**		**Adjusted[a]**		**p value**
			**Odds Ratio**	**(95% CI)**	**Odds Ratio**	**(95% CI)**	
**Age group**	<1	67 (6.8)	0.19	(0.06-0.62)	**0.24**	**(0.06-0.92)**	**0.04**
	1-4	456 (46.1)	1.0 (reference)		1.0 (reference)		
	5-9	274 (27.7)	0.62	(0.40-0.94)	**0.45**	**(0.26-0.76)**	**0.003**
	10-15	192 (19.4)	0.34	(0.19-0.61)	**0.22**	**(0.11-0.47)**	**<0.001**
**Sex**	Male	513 (51.9)	1.0 (reference)		1.0 (reference)		
	Female	476 (48.1)	1.37	(0.96-1.96)	1.40	(0.91-2.16)	0.13
**Travel**	No	850 (86.0)	1.0 (reference)		1.0 (reference)		
	Yes	139 (14.0)	0.46	(0.24-0.88)	0.58	(0.26-1.28)	0.18
**Rurality**	Urban	719 (72.7)	1.0 (reference)		1.0 (reference)		
	Rural	270 (27.3)	1.21	(0.82-1.77)	0.97	(0.58-1.62)	0.90
**IMD Quintile**	1 (least disadvantaged)	231 (23.4)	1.0 (reference)		1.0 (reference)		
	2	210 (21.2)	0.83	(0.48-1.42)	0.65	(0.33-1.27)	0.21
	3	204 (20.6)	1.28	(0.77-2.12)	0.98	(0.51-1.79)	0.89
	4	170 (17.2)	1.10	(0.64-1.89)	0.96	(0.48-1.94)	0.91
	5 (most disadvantaged)	174 (17.6)	0.57	(0.30-1.06)	**0.41**	**(0.19-0.90)**	**0.03**
**Region**	East Midlands	72 (7.3)	0.62	(0.24-1.59)	0.65	(0.21-2.02)	0.45
	East of England	66 (6.7)	1.03	(0.44-2.42)	1.37	(0.47-4.00)	0.56
	London	108 (10.9)	1.0 (reference)		1.0 (reference)		
	North East	76 (7.7)	1.19	(0.53-2.64)	1.02	(0.38-2.71)	0.97
	North West	185 (18.7)	1.20	(0.63-2.31)	1.29	(0.57-2.90)	0.54
	South East	107 (10.8)	1.09	(0.52-2.28)	1.74	(0.65-4.63)	0.27
	South West	127 (12.8)	1.48	(0.75-2.93)	1.64	(0.68-3.99)	0.27
	West Midlands	104 (10.5)	0.54	(0.23-1.29)	0.67	(0.24-1.86)	0.44
	Yorkshire and Humber	144 (14.6)	0.62	(0.29-1.33)	0.60	(0.24-1.52)	0.28
Stx	*Stx*1+2	226 (22.9)	1.0 (reference)		1.0 (reference)		
	*Stx*1	18 (1.8)	1.84	(0.21-15.84)	5.34	(0.54-52.82)	0.15
	*Stx*2	745 (75.3)	6.99	(3.22-15.17)	**5.76**	**(2.43-13.67)**	**<0.001**
**Antibiotics**	No	887 (89.7)	1.0 (reference)		1.0 (reference)		
	Yes	102 (10.3)	8.54	(5.48-13.30)	**7.46**	**(4.27-13.03)**	**<0.001**
**Diarrhea**	No	49 (5.0)	1.0 (reference)		1.0 (reference)		
	Yes	940 (95.0)	8.61	(1.18-62.89)	4.09	(0.51-32.47)	0.18

Confidential: For Review Only

**Bloody diarrhea**	No	440 (44.5)	1.0 (reference)		1.0 (reference)		
	Yes	549 (55.5)	4.85	(3.07-7.67)	**3.74**	**(2.15-6.49)**	**<0.001**
**Nausea**	No	653 (66.0)	1.0 (reference)		1.0 (reference)		
	Yes	336 (34.0)	1.52	(1.06-2.18)	1.11	(0.67-1.83)	0.69
**Vomiting**	No	549 (55.5)	1.0 (reference)		1.0 (reference)		
	Yes	440 (44.5)	6.05	(3.95-9.26)	**4.38**	**(2.59-7.40)**	**<0.001**
**Abdominal pain**	No	309 (31.2)	1.0 (reference)		1.0 (reference)		
	Yes	680 (68.8)	1.49	(0.99-2.25)	0.83	(0.47-1.46)	0.52
**Fever**	No	657 (66.4)	1.0 (reference)		1.0 (reference)		
	Yes	332 (33.6)	1.50	(1.05-2.16)	1.04	(0.66-1.63)	0.86

aAdjusted for all other covariates in the model; *stx* – shiga toxin gene

Confidential: For Review Only

Supplementary Figure 1: Selection of participants to HUS Cohort Study

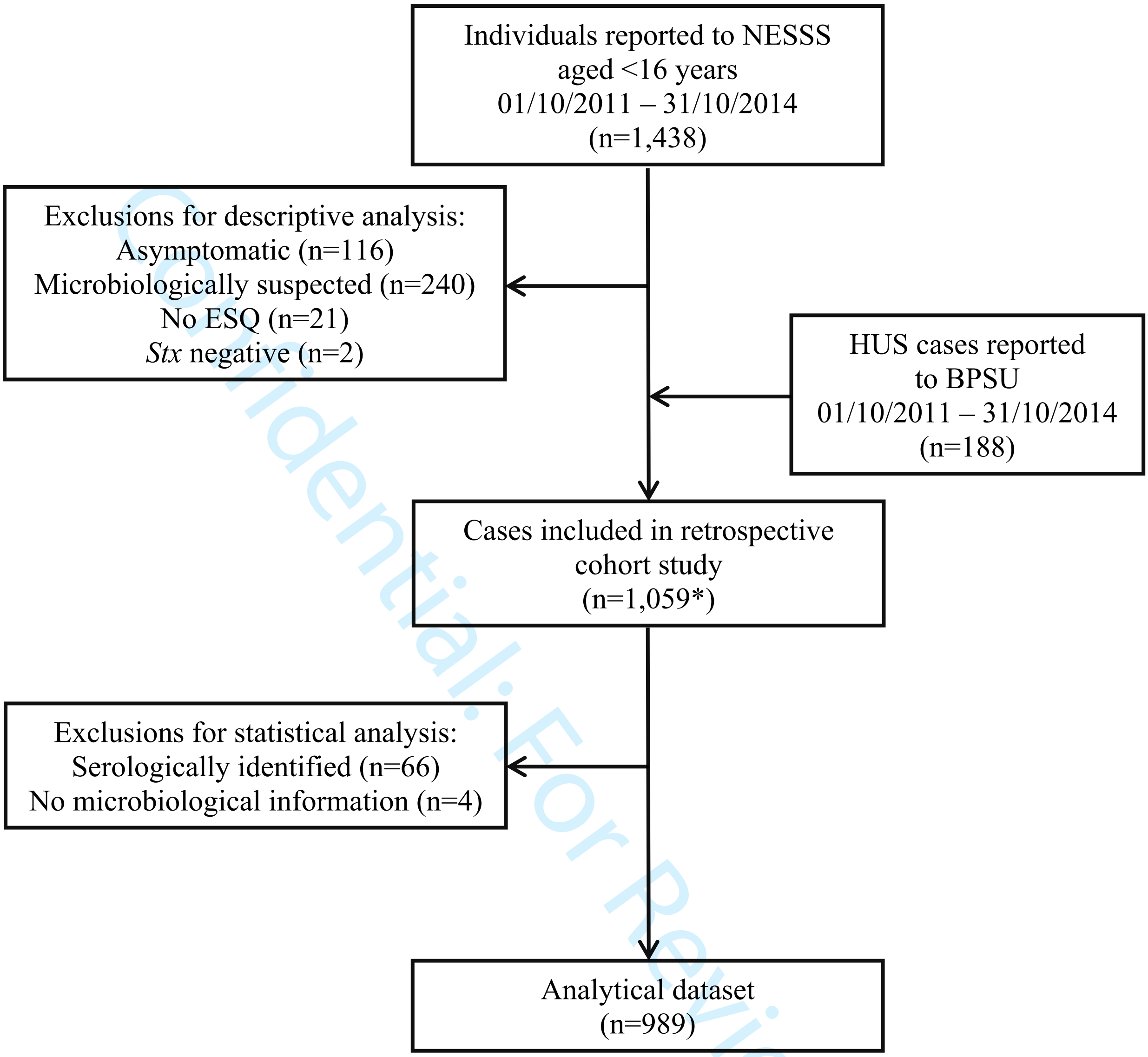

*An additional 19 HUS cases not reported to BPSU were identified in NESSS; NESSS – National Enhanced Surveillance System for STEC; HUS – haemolytic uraemic syndrome; BPSU – British Paediatric Surveillance Unit; ESQ – enhanced surveillance questionnaire

Confidential: For Review Only

STROBE Statement—Checklist of items that should be included in reports of ***cohort studies***

	Item No	Recommendation
**Title and abstract**	1	(*a*) Indicate the study's design with a commonly used term in the title or the abstract
		(*b*) Provide in the abstract an informative and balanced summary of what was done and what was found
**Introduction**		
Background/rationale	2	Explain the scientific background and rationale for the investigation being reported
Objectives	3	State specific objectives, including any prespecified hypotheses
**Methods**		
Study design	4	Present key elements of study design early in the paper
Setting	5	Describe the setting, locations, and relevant dates, including periods of recruitment, exposure, follow-up, and data collection
Participants	6	(*a*) Give the eligibility criteria, and the sources and methods of selection of participants. Describe methods of follow-up
		(*b*) For matched studies, give matching criteria and number of exposed and unexposed
Variables	7	Clearly define all outcomes, exposures, predictors, potential confounders, and effect modifiers. Give diagnostic criteria, if applicable
Data sources/ measurement	8*	For each variable of interest, give sources of data and details of methods of assessment (measurement). Describe comparability of assessment methods if there is more than one group
Bias	9	Describe any efforts to address potential sources of bias
Study size	10	Explain how the study size was arrived at
Quantitative variables	11	Explain how quantitative variables were handled in the analyses. If applicable, describe which groupings were chosen and why
Statistical methods	12	(*a*) Describe all statistical methods, including those used to control for confounding
		(*b*) Describe any methods used to examine subgroups and interactions
		(*c*) Explain how missing data were addressed
		(*d*) If applicable, explain how loss to follow-up was addressed
		(*e*) Describe any sensitivity analyses
**Results**		
Participants	13*	(a) Report numbers of individuals at each stage of study—eg numbers potentially eligible, examined for eligibility, confirmed eligible, included in the study, completing follow-up, and analysed
		(b) Give reasons for non-participation at each stage
		(c) Consider use of a flow diagram
Descriptive data	14*	(a) Give characteristics of study participants (eg demographic, clinical, social) and information on exposures and potential confounders
		(b) Indicate number of participants with missing data for each variable of interest
		(c) Summarise follow-up time (eg, average and total amount)
Outcome data	15*	Report numbers of outcome events or summary measures over time
Main results	16	(*a*) Give unadjusted estimates and, if applicable, confounder-adjusted estimates and their precision (eg, 95% confidence interval). Make clear which confounders were adjusted for and why they were included
		(*b*) Report category boundaries when continuous variables were categorized
		(*c*) If relevant, consider translating estimates of relative risk into absolute risk for a meaningful time period

Confidential: For Review Only

Other analyses	17	Report other analyses done—eg analyses of subgroups and interactions, and sensitivity analyses
**Discussion**		
Key results	18	Summarise key results with reference to study objectives
Limitations	19	Discuss limitations of the study, taking into account sources of potential bias or imprecision. Discuss both direction and magnitude of any potential bias
Interpretation	20	Give a cautious overall interpretation of results considering objectives, limitations, multiplicity of analyses, results from similar studies, and other relevant evidence
Generalisability	21	Discuss the generalisability (external validity) of the study results
**Other information**		
Funding	22	Give the source of funding and the role of the funders for the present study and, if applicable, for the original study on which the present article is based

*Give information separately for exposed and unexposed groups.

Note: An Explanation and Elaboration article discusses each checklist item and gives methodological background and published examples of transparent reporting. The STROBE checklist is best used in conjunction with this article (freely available on the Web sites of PLoS Medicine at http://www.plosmedicine.org/, Annals of Internal Medicine at http://www.annals.org/, and Epidemiology at http://www.epidem.com/). Information on the STROBE Initiative is available at http://www.strobe-statement.org.

Confidential: For Review Only

BMJ Paediatrics Open

Sociodemographic and clinical risk-factors for paediatric typical haemolytic uraemic syndrome

Journal:	*BMJ Paediatrics Open*
Manuscript ID	bmjpo-2019-000465.R2
Article Type:	Original article
Date Submitted by the Author:	07-Aug-2019
Complete List of Authors:	Adams, Natalie; NIHR Health Protection Research Unit in Gastrointestinal Infections; Public Health England, National Infection Service<br>Byrne, Lisa; Public Health England, National Infection Service<br>Rose, Tanith; NIHR Health Protection Research Unit in Gastrointestinal Infections; University of Liverpool, Department of Public Health and Policy<br>Adak, Bob; NIHR Health Protection Research Unit in Gastrointestinal Infections<br>Jenkins, Claire; Public Health England, National Infection Service; NIHR Health Protection Research Unit in Gastrointestinal Infections<br>Charlett, Andre; Public Health England, National Infection Service<br>Violato, Mara; NIHR Health Protection Research Unit in Gastrointestinal Infections; University of Oxford, Health Economics Research Centre<br>O'Brien, Sarah; NIHR Health Protection Research Unit in Gastrointestinal Infections; University of Liverpool, Department of Public Health and Policy<br>Whitehead, Margaret; NIHR Health Protection Research Unit in Gastrointestinal Infections; University of Liverpool, Department of Public Health and Policy<br>Barr, Benjamin; NIHR Health Protection Research Unit in Gastrointestinal Infections; University of Liverpool, Department of Public Health and Policy<br>Taylor-Robinson, David; NIHR Health Protection Research Unit in Gastrointestinal Infections; University of Liverpool, Department of Public Health and Policy<br>Hawker, Jeremy; NIHR Health Protection Research Unit in Gastrointestinal Infections; Public Health England, National Infection Service
Keywords:	Epidemiology, Infectious Diseases

SCHOLARONE™
Manuscripts

Title Page

Title: Sociodemographic and clinical risk-factors for paediatric typical haemolytic uraemic syndrome: a retrospective cohort study

Authors:

Natalie L. Adams*[1,2,3,], Lisa Byrne[1,3], Tanith C. Rose[1,2], Goutam K. Adak[1], Claire Jenkins[1,3], Andre Charlett[3], Mara Violato[1,4], Sarah J. O'Brien[1,2], Margaret Whitehead[1,2], Benjamin Barr[1,2], David Taylor-Robinson[1,2±] and Jeremy Hawker[1,3±]

*Corresponding author

± Joint senior authors

Affiliations:

[1]NIHR Health Protection Research Unit in Gastrointestinal Infections, UK

[2]Department of Public Health and Policy, University of Liverpool, UK

[3]National Infection Service, Public Health England, UK

[4]Health Economics Research Centre, University of Oxford, Oxford, UK

Disclaimers:

The research was funded by the National Institute for Health Research Health Protection Research Unit (NIHR HPRU) in Gastrointestinal Infections at University of Liverpool in partnership with Public Health England (PHE), in collaboration with University of East Anglia, University of Oxford and the Quadram Institute. Natalie Adams is based at the University of Liverpool and Public Health England. The views expressed are those of the

Confidential: For Review Only

author(s) and not necessarily those of the NHS, the NIHR, the Department of Health or Public Health England.

Running title:

Paediatric HUS in England 2011-2014

Corresponding author contact information:

Dr Natalie L Adams, Gastrointestinal Infections Department, National Infection Service, Public Health England, 61 Colindale Ave, Colindale NW9 5EQ, UK

Natalie.Adams@phe.gov.uk

Word count (abstract): 243/250

Word count (text): 2841/2500

References: 32/40

Confidential: For Review Only

ABSTRACT

Objectives

Haemolytic Uraemic Syndrome (HUS) following Shiga toxin-producing *Escherichia coli* (STEC) infection is the commonest cause of acute renal failure among children in the United Kingdom. This study explored differential progression from STEC to HUS by social, demographic and clinical risk factors.

Methods

We undertook a retrospective cohort study linking two datasets. We extracted data on paediatric STEC and HUS cases identified in the Public Health England National Enhanced Surveillance System for STEC and British Paediatric Surveillance Unit HUS surveillance from October 1 2011 to October 31 2014. Using logistic regression, we estimated the odds of HUS progression by risk factors.

Results

1059 paediatric STEC cases were included in the study, of which 207 (19.55%, 95% CI 17-22%) developed HUS. In the fully-adjusted model, the odds of progression to HUS were highest in those aged 1-4 (OR 4.93, 95% CI 2.30-10.56, compared to 10-15 years), were infected with an stx2-only strain (OR 5.92, 95%CI 2.49-14.10), were prescribed antibiotics (OR 8.46, 95% CI 4.71-15.18), had bloody diarrhoea (OR 3.56, 95%CI 2.04-6.24) or vomiting (OR 4.47, 95%CI 2.62-7.63), but there was no association with progression to HUS by socioeconomic circumstances or rurality.

Conclusion

Confidential: For Review Only

Combining data from an active clinical surveillance system for HUS with the national enhanced STEC surveillance system suggests that 20% of diagnosed paediatric STEC infections in England resulted in HUS No relationship was found with socioeconomic status or rurality of cases, but differences were demonstrated by age, stx type and presenting symptoms.

Keywords: Health inequalities; Shiga-toxin producing *Escherichia* coli; STEC; Haemolytic Uraemic Syndrome; HUS

Confidential: For Review Only

INTRODUCTION

Haemolytic uraemic syndrome (HUS) is a rare but serious complication of infection with Shiga toxin-producing *Escherichia coli* (STEC), affecting the blood, kidneys and, in the most severe cases, the central nervous system. Children and the elderly are considered to be the most susceptible age groups and HUS is recognised as the most common cause of acute renal failure among children in the United Kingdom (1). Strains of STEC encoding s*tx2* toxin genes are more often associated with HUS than other strains (1-6). STEC serogroup O157 is the most frequently reported strain causing illness in England. Transmission to humans occurs through consumption of contaminated food or water, exposure to a contaminated environment involving direct or indirect contact with animals or their faeces and person-to-person spread.

It is estimated that progression to HUS following STEC infection could be as high as 15% in young children (2, 7). Several studies have suggested that development of HUS varies by some demographic characteristics; higher incidence of HUS has been documented in children (particularly aged 1-4), females (particularly aged over 10 years) and in those of White ethnicity (2, 6, 8-13); however few have documented progression to HUS by other demographic characteristics such as deprivation, foreign travel, rurality or region. There is evidence to suggest that those who are disadvantaged have a lower risk of STEC infection (14-16), and potentially a lower risk of progression to HUS outside of England (16,17), however no studies have looked at the relationship between SES, STEC and HUS in England. This study aims to investigate the relationship between demographic factors, STEC infection and subsequent development of HUS in a well-characterised paediatric population in England with high case-ascertainment.

METHODS

Confidential: For Review Only

Data, setting and source

We undertook a retrospective cohort study linking two data sources; the Public Health England (PHE) National Enhanced Surveillance System for STEC (NESSS) and the British Paediatric Surveillance Unit (BPSU) HUS Study in conjunction with PHE. The linkage of two robust datasets, both of which can record HUS status, ensures high ascertainment of HUS cases. Firstly, we extracted data on STEC cases aged 0-15 years (inclusive) identified in NESSS during the period of the BPSU HUS Study (October 1 2011 to October 31 2014). All laboratory-confirmed STEC cases in England are reported by NHS laboratories to Public Health England staff who collect standardised data through an enhanced surveillance questionnaire (ESQ) as part of their public health response: this standardised dataset is collated centrally in NESSS for further validation and analysis. The ESQ collects detailed information on patient demographics, symptoms, food and water exposures and UK and non-UK travel during the exposure period (the week prior to illness onset). When a presumptive STEC is identified at the local laboratory or a case of HUS is identified, specimens are sent to PHE Gastrointestinal Bacteria Reference Unit (GBRU) for testing and patient ESQs are linked to microbiological results. Due to the timing of the ESQ administration in NESSS (which is designed to inform the acute public health response), this system can under-ascertain HUS as this can develop after completion of the questionnaire. This surveillance system is described in detail elsewhere (2).

Secondly, we extracted clinical data on paediatric (<16 years) HUS cases, collected by the BPSU HUS Study, an active surveillance system requiring regular returns from clinicians. Within this study, data were captured using a standardised questionnaire administered to paediatricians collecting information on; case demography; treatment history; microbiological investigations; clinical parameters of illness; clinical management of illness and status of the case at the time of data capture. Cases in the BPSU dataset were linked on

Confidential: For Review Only

National Health Service (NHS) number, which was available for all cases, to those in the NESSS dataset to create a retrospective cohort. Supplementary Figure 1 provides details of the selection of study participants.

For statistical analysis, cases for whom no microbiological information was available (n=4) and cases identified via serological testing only (n=66) were excluded in order to assess the role of *stx* subtype. Ethnic groups, collected in five categories (White, Asian/Asian British, Black/Black British, Mixed, Chinese) is not well-completed in NESSS and therefore responses were re-coded as White or non-White for analysis. The considerable missing data for the ethnicity variable (19.1%) has led us to use the crude dichotomy of White/non-White in this analysis. Multiple imputation using chained equations to impute values where ethnicity (White/non-White) was missing. There will clearly be some loss of information from doing this, and this precludes investigating risk differences between the non-White ethnic groups . This may also slightly affect the confounding that exists between ethnicity and socioeconomic status. Fifty imputed datasets were generated. The distribution of ethnicity by age, sex and region was assessed to check the missing at random (MAR) assumption. There was no difference in missing ethnicity by sex, however there were some differences by age group (57.3% of cases with missing ethnicity were in the 1-4 age group; n=114/199) and region (31.2% of cases with missing ethnicity were in London; n=62/199); these were not regarded as problematic however as, given the observed data for other variables, the missing data were considered independent.

Ethics

Ethical approval was originally obtained for the main study (Ref: 11/LO/1412). As of October 2010, HUS is a statutory reportable condition and this study falls under the existing Health Protection Agency (now Public Health England) permissions under Section 251 of the

Confidential: For Review Only

NHS Act 2006. In addition, we received a favourable ethical opinion from the South East Coast - Surrey Research Ethics Committee (15/LO/2138) on 1 December 2015 covering the use of this dataset for this study.

Patient involvement

Patients were not directly involved in the design of this study.

Outcome and exposures

The outcome of interest was HUS, determined by the case meeting the BPSU clinical criteria (See supplementary Table 1) (18) or completion of the HUS field in the ESQ. Covariates in the analysis were age group (<1, 1-4, 5-9, 10-15 years); sex (male/female); ethnicity (White/non-White); travel (yes/no); rurality (rural/urban); microbiology (Shiga-toxin (*Stx*)); antibiotic use (yes/no); clinical symptoms (diarrhoea, bloody diarrhoea, nausea, vomiting, abdominal pain, fever) and region of residence. The s*tx* type, the primary STEC virulence factor, was used as the main microbiological variable (19). Where symptoms, travel status and healthcare contact variables were blank or unknown, these were recoded as a negative response. As a proxy for childhood socioeconomic circumstances (SECs) we used a small-area deprivation measure, the Index of Multiple Deprivation 2010 (IMD) (20), assigned to each case based on their postcode and divided into population-level quintiles, with the first quintile representing the least deprived and the fifth quintile representing the most deprived.

Analysis strategy

Comparisons of proportions were tested using the chi-squared test. We explored univariate relationships between progression to HUS and the covariates of interest before fitting a multivariate logistic regression model. All variables were retained in this model in order to control for any potential confounding. Interaction terms between variables (IMD, ethnicity,

https://mc.manuscriptcentral.com/bmjpo

Confidential: For Review Only

age and sex) were tested to investigate whether the strength of any relationship was moderated by the inclusion of another variable. Analyses were conducted in Stata 13.1 (Statacorp, Texas).

Robustness tests

We performed multiple sensitivity analyses to test the validity of the main analysis; first by (i) excluding cases that were likely to have a travel-acquired STEC infection (date of onset is within one exposure period, 7 days, of having returned from outside of the UK) and (ii) separately excluding cases with unknown ethnicity to determine whether there were differences in progression to HUS by SECs for children who travelled abroad during their incubation period compared to those who did not or those with ethnicity recorded and those without respectively.

RESULTS

Descriptive analysis

Of 1059 paediatric STEC cases included in the study, 207 (19.55%, 95% CI 17.27%-22.04%) developed HUS. Progression to HUS varied by age and gender (Table 1), the highest was observed in females aged 1-4 years (26.0%). A higher proportion of progression to HUS was observed in females aged 10-15 years compared to males of the same age (19.3%, 95% CI 12.3-27.9 versus 7.1%, 95% CI 2.9-14.2, p=0.01), and amongst females aged less than 1 year compared to males of the same age, although this was not significant (14.3%, 95% CI 4.0-32.7 versus 4.8%, 95% CI 0.6-16.2, p=0.16). Although progression to HUS was higher in the least disadvantaged quintile (47/245, 19.2%, 95% CI 14.4-24.7%) compared with the most disadvantaged quintile (29/189, 15.3%, 95% CI 10.5-21.3%) this difference was not statistically significant (p=0.29). The highest proportion progressing to HUS was in quintile 3

Confidential: For Review Only

(53/219, 24.2%, 95% CI 18.7-30.4%) and there was no clear pattern across the 5 quintiles (p=0.07; quintile 2 - 35/221, 15.8%, 95% CI 11.3-21.3%; quintile 4 - 43/185, 23.2%, 95% CI 17.4-30%).

Multivariable analysis

In the fully adjusted model (Table 2), there were significantly lower odds of HUS amongst <1, 5-9 and 10-15 year olds compared to 1-4 year olds and significantly higher odds of HUS amongst those infected with *stx*2-only strains, those prescribed antibiotics and among those who had experienced bloody diarrhoea or vomiting . The most disadvantaged children had lower odds of progression to HUS compared to the least disadvantaged children (OR 0.57, 95% CI 0.25-1.31) but the difference was not significant. There was no statistically significant difference in risk by rurality (OR 0.88, 95% CI 0.52-1.48) or by region (Table 2). There were no significant interactions identified (data not shown).

The sensitivity analyses conducted to assess the robustness of the findings did not alter the overall conclusions of this research (Supplementary Tables 2 and 3).

DISCUSSION

In a novel linkage and analysis of two datasets with high case-ascertainment to explore the role of demographic and socioeconomic factors in the development of HUS following STEC infection, we found progression from STEC infection to HUS to be 20% in this paediatric cohort in England. Odds of HUS progression varied by age, stx type, antibiotic exposure and clinical presentation, with children aged 1-4 years infected with stx2-only, with reported antibiotic exposure and presenting with bloody diarrhoea or vomiting at highest risk. Few studies have explored the social patterning of risk factors for STEC (21) or the socio-demographic risk factors associated with progression to HUS, and no such studies have been

Confidential: For Review Only

undertaken in England. We found no relationship between progression to HUS and socioeconomic status in children in this study.

Our study has several strengths. This study captures the progression of HUS in a well-characterised paediatric STEC population. To the best of our knowledge, as confirmed by a prior review of the literature and discussion with national experts, this is the first study to combine a prospective active surveillance system and a multisource national surveillance system to study the risk factors for HUS and as such is likely to have better case-ascertainment of HUS than previous studies and is related to good STEC denominators. Furthermore, this study makes use of one of the largest cohorts of HUS cases. The results of this study are likely to be generalisable to other high-income countries with a similar pattern of STEC infection. Despite this, there are some limitations. It is possible that there is residual confounding that could not be controlled for, such as intrinsic childhood characteristics which may increase differential vulnerability or susceptibility by SEC such as genetic predisposition, co-morbidities, and clinical or treatment characteristics. Further, as an area-level measure of SEC was used, it is possible that it may not have been sensitive enough to detect the effect of socioeconomic inequalities, particularly if individual factors rather than area-level factors have more influence over the risk of acquiring more severe strains of STEC with increased risk of progression to HUS. However, person-to-person spread is an important risk factor for GI infections and, although there is a risk of ecological fallacy, area-level measures have the advantage of including potential environmental factors such as housing and living environment deprivation which are likely to be important factors in considering individual risk of infection. Excluding individuals with a serological result only from the statistical analysis may introduce a potential bias leading to an underestimate of HUS incidence, which may be important if there are geographical or host-factors which are linked to severity of illness, although the number of serology-only diagnoses was small. In England,

Confidential: For Review Only

most diagnosed cases of STEC are of serogroup O157 (95% in our study), and it is possible that our results may be biased towards the relationship between STEC O157 and progression to HUS, which may differ if other, possibly less pathogenic, serogroups predominate. It is possible however, that the risk of progression to HUS could be different in populations exposed to STEC organisms with a lower proportion of stx2-only producing strains, or with a different age distribution of cases. There were also some missing data in our study, particularly for ethnicity, which we addressed using multiple imputation. The binary ethnicity variable used (White/non-White) was also crude and adopted because of data quality issues in NESSS for this variable. However, a previous study using this data (2) demonstrated differences in risk of STEC between White and non-White ethnic groups (RR 1.43, p<0.001) and so was important to assess in our study although its inclusion may mask differences in socioeconomic status. No data were available on whether the children included in our study had underlying or chronic conditions which may be related to their risk of developing HUS. Finally, it was not always possible to determine whether antibiotics had been prescribed during treatment for STEC infection or following a diagnosis of HUS therefore the relevant association should be interpreted with caution.

The finding of 19.5% (95% CI 17-22%) of diagnosed STEC cases progressing to HUS is higher than previous studies, which have estimated the proportion of paediatric cases of STEC O157 progressing to HUS to be 15% (95% CI 11-19%) in females aged 1-4 years in England (2) and 15.3% (95% CI 13-18%) in children aged <5 years in the USA (7). Our study uses data derived from two linked surveillance systems providing high ascertainment of both STEC and HUS cases which provides a more robust estimate. It is likely that there will also be a bias resulting from ascertainment of STEC cases from laboratory specimens, as milder cases of gastrointestinal infection are less likely to be microbiologically tested, but this will also be true of previous published studies.

Confidential: For Review Only

Whilst rurality has been reported as an important factor in risk for STEC infection (2, 14), our study suggests that rurality is not a significant driver of progression to HUS. It is important to note that there are environmental factors, such as cattle density, that were not included in this study and which may be more important factors in risk of STEC infection. Our finding that rurality was not linked to progression to HUS following STEC infection may also be due to the majority of our cases (95%) being STEC O157 – this finding may be different in more heterogenous dataset from countries with greater variability by serogroup. Similarly, despite evidence to suggest that the risk and consequences of GI infections in general are greater for disadvantaged children (22-26) – the finding in our study suggests that lower childhood SEC is unlikely to be a contributor for development of HUS.

Previous studies in England have suggested that children aged 1-4 years, females and white ethnic groups have the highest incidence of STEC infection (2, 27). Our study echoes the findings by Milford et al (6) which demonstrated higher progression to HUS amongst children aged 1-4 years. No overall difference in risk of HUS by sex was identified in our study, a finding echoed in several other previous studies (28-31); this is an area of disagreement in the literature with several studies finding higher risk amongst women (7, 17, 32) although two of these studies finding higher risk in women did not look specifically among children (7, 32). We did find differences in risk by sex within specific age groups, with a greater proportion of progression to HUS amongst girls less than 1 year of age and 10-15 years of age compared to boys of the same age groups (Table 1), although no significant interaction between age and sex could be identified. The reasons for the differential risk by age are currently unclear and call for a deeper understanding of differences in risks and exposures between these groups.

The association between clinical presentation with vomiting and bloody diarrhoea and increased risk of HUS reported in this study has been identified previously (12) and, as such,

https://mc.manuscriptcentral.com/bmjpo

Confidential: For Review Only

the presence of these symptoms particularly in paediatric STEC cases should evoke a high level of clinical suspicion for the potential development of HUS.

Our study quantifies the proportion of paediatric STEC cases progressing to HUS in a well – defined population with high ascertainment. It also quantifies the risk factors associated with progression to HUS in terms of sociodemographic characteristics as well as clinical presentation. Further research is warranted to elucidate the populations at risk of STEC infection and HUS in terms of deprivation, ethnicity, age and sex, in order to better understand whether there are real differences in risk or artefacts of surveillance.

FUNDING

The research was funded by the National Institute for Health Research Health Protection Research Unit (NIHR HPRU) in Gastrointestinal Infections [grant number NIHR HPRU 201210,038] at University of Liverpool in partnership with Public Health England (PHE), in collaboration with University of East Anglia, University of Oxford and the Quadram Institute. Natalie Adams is based at the University of Liverpool and Public Health England. The views expressed are those of the author(s) and not necessarily those of the NHS, the NIHR, the Department of Health and Social Care or Public Health England. David Taylor-Robinson is funded by the MRC on a Clinician Scientist Fellowship (MR/P008577/1). Ben Barr and Tanith Rose were also supported by the National Institute for Health Research (NIHR) Collaboration for Leadership in Health Research and Care (CLAHRC NWC).

CONFLICT OF INTEREST

All authors: No potential conflicts of interest.

ACKNOWLEDGMENTS

Confidential: For Review Only

This work was funded by the National Institute for Health Research Health Protection Research Unit in Gastrointestinal Infections. The authors would like to thank Kirsten Glen and Naomi Launders for their work on the National Enhanced Surveillance System for STEC, Ross Harris for statistical advice and Richard Lynn at the British Paediatric Surveillance Unit for facilitating the BPSU HUS study.

CONFLICT OF INTEREST

The authors declare that they have no conflicts of interest.

CONTRIBUTORSHIP STATEMENT

All authors contributed to the conception and design of the study. LB, CJ and GK collated and curated the dataset and provided guidance on interpretation of data. NA performed the analyses with guidance from AC, LB, BB, JH and DTR. NA, JH, DTR, MV, SJOB and MM drafted the manuscript which was critically revised by all authors. All authors approved the final version of the manuscript. NA submitted the manuscript. JH and DTR are joint senior authors.

Confidential: For Review Only

WHAT IS ALREADY KNOWN ON THIS TOPIC

- HUS is recognised as the most common cause of acute renal failure among children in the United Kingdom.
- It is estimated that progression to HUS following STEC infection could be as high as 15% in young children.
- Several studies have suggested that development of HUS varies by demographic characteristics however few have documented progression to HUS by demographic characteristics.

WHAT THIS STUDY ADDS

- A fifth of paediatric STEC cases developed the serious complication of haemolytic uraemic syndrome in England.
- This figure is higher than previously reported in England, and varied by demographic and clinical factors
- Socioeconomic factors did not influence progression to SES.

Confidential: For Review Only

REFERENCES

1. Lynn RM, O'Brien SJ, Taylor CM, et al. Childhood hemolytic uremic syndrome, United Kingdom and Ireland. Emerging Infectious Diseases. 2005;11(4):590-6. doi:10.3201/eid1104.040833
2. Byrne L, Jenkins C, Launders N, Elson R, Adak GK. The epidemiology, microbiology and clinical impact of Shiga toxin-producing Escherichia coli in England, 2009-2012. Epidemiology and Infection. 2015:1-13. doi:10.1017/S0950268815000746
3. Ethelberg S, Olsen KE, Scheutz F, et al. Virulence factors for hemolytic uremic syndrome, Denmark. Emerging Infectious Diseases. 2004;10(5):842-7. doi:10.3201/eid1005.030576
4. Persson S, Olsen KE, Ethelberg S, Scheutz F. Subtyping method for Escherichia coli shiga toxin (verocytotoxin) 2 variants and correlations to clinical manifestations. J Clin Microbiol. 2007;45(6):2020-4. doi:10.1128/JCM.02591-06
5. Dallman TJ, Ashton PM, Byrne L, et al. Applying phylogenomics to understand the emergence of Shiga-toxin-producing Escherichia coli O157:H7 strains causing severe human disease in the UK. Microbial Genomics. 2015;1(3). doi:doi:10.1099/mgen.0.000029
6. Milford DV, Taylor CM, Guttridge B, Hall SM, Rowe B, Kleanthous H. Haemolytic uraemic syndromes in the British Isles 1985-8: association with verocytotoxin producing Escherichia coli. Part 1: Clinical and epidemiological aspects. Archives of Disease in Childhood. 1990;65(7):716-21.
7. Gould LH, Demma L, Jones TF, et al. Hemolytic uremic syndrome and death in persons with Escherichia coli O157:H7 infection, foodborne diseases active surveillance network sites, 2000-2006. Clinical Infectious Diseases. 2009;49(10):1480-5. doi:10.1086/644621
8. Kinney JS, Gross TP, Porter CC, Rogers MF, Schonberger LB, Hurwitz ES. Hemolytic-uremic syndrome: a population-based study in Washington, DC and Baltimore, Maryland. American Journal of Public Health. 1988;78(1):64-5.
9. Rogers MF, Rutherford GW, Alexander SR, et al. A population-based study of hemolytic-uremic syndrome in Oregon, 1979-1982. American Journal of Epidemiology. 1986;123(1):137-42.
10. Tarr PI, Hickman RO. Hemolytic uremic syndrome epidemiology: a population-based study in King County, Washington, 1971 to 1980. Pediatrics. 1987;80(1):41-5.
11. Bell WR. Thrombotic thrombocytopenic purpura/hemolytic uremic syndrome relapse: frequency, pathogenesis, and meaning. Seminars in hematology. 1997;34(2):134-9.
12. Launders N, Byrne L, Jenkins C, Harker K, Charlett A, Adak GK. Disease severity of Shiga toxin-producing E. coli O157 and factors influencing the development of typical haemolytic uraemic syndrome: a retrospective cohort study, 2009–2012. BMJ open. 2016;6(1). doi:10.1136/bmjopen-2015-009933
13. Elliott EJ, Robins-Browne RM, O'Loughlin EV, et al. Nationwide study of haemolytic uraemic syndrome: clinical, microbiological, and epidemiological features. Arch Dis Child. 2001;85:125-31.
14. Chang M, Groseclose SL, Zaidi AA, Braden CR. An ecological analysis of sociodemographic factors associated with the incidence of salmonellosis, shigellosis, and E. coli O157:H7 infections in US counties. Epidemiology and infection. 2009;137(6):810-20. doi:10.1017/s0950268808001477
15. Jalava K, Ollgren J, Eklund M, Siitonen A, Kuusi M. Agricultural, socioeconomic and environmental variables as risks for human verotoxigenic Escherichia coli (VTEC) infection in Finland. BMC Infect Dis. 2011;11:275. doi: http://dx.doi.org/10.1186/1471-2334-11-275. PubMed PMID: 22008456; PubMed Central PMCID: PMCPMC3226588.

https://mc.manuscriptcentral.com/bmjpo

16. Whitney BM, Mainero C, Humes E, Hurd S, Niccolai L, Hadler JL. Socioeconomic status and foodborne pathogens in Connecticut, USA, 2000–20111. Emerging Infectious Diseases. 2015;21(9):1617-24. doi: 10.3201/eid2109.150277.
17. Rowe PC, Orrbine E, Wells GA, McLaine PN. Epidemiology of hemolytic-uremic syndrome in Canadian children from 1986 to 1988. The Canadian Pediatric Kidney Disease Reference Centre. Journal of Pediatrics. 1991;119(2):218-24.
18. Byrne L, on behalf of the BPSU HUS Study Team,. BPSU HUS Study Report. 2017.
19. Ethelberg S, Olsen KE, Scheutz F, et al. Virulence factors for hemolytic uremic syndrome, Denmark. Emerg Infect Dis. 2004;10(5):842-7. doi:10.3201/eid1005.030576
20. Department for Communities and Local Government. English Indices of Deprivation 2010. Department for Communities and Local Government. 2011. https://www.gov.uk/government/uploads/system/uploads/attachment_data/file/6871/1871208.pdf. Accessed 16/09/2016.
21. Bentancor AB, Ameal LA, Calvino MF, Martinez MC, Miccio L, Degregorio OJ. Risk factors for Shiga toxin-producing Escherichia coli infections in preadolescent schoolchildren in Buenos Aires, Argentina. Journal of Infection in Developing Countries. 2012;6(5):378-86.
22. Olowokure B, Hawker J, Weinberg J, Gill N, Sufi F. Deprivation and hospital admission for infectious intestinal diseases. Lancet. 1999;353(9155):807-8. doi:10.1016/S0140-6736(99)00611-X
23. Phillips G, Tam CC, Rodrigues LC, Lopman B. Risk factors for symptomatic and asymptomatic norovirus infection in the community. Epidemiology and Infection. 2011;139(11):1676-86. doi:http://dx.doi.org/10.1017/S0950268810002839
24. Pockett RD, Adlard N, Carroll S, Rajoriya F. Paediatric hospital admissions for rotavirus gastroenteritis and infectious gastroenteritis of all causes in England: an analysis of correlation with deprivation. Current medical research and opinion. 2011;27(4):777-84. doi:10.1185/03007995.2011.555757
25. Rose TC, Adams NL, Barr B, et al. Socioeconomic status is associated with symptom severity and sickness absence in people with infectious intestinal disease in the UK. BMC Infect Dis. 2017;17(1):447. doi:10.1186/s12879-017-2551-1
26. Adams NL, Rose TC, Hawker J, et al. Relationship between socioeconomic status and gastrointestinal infections in developed countries: A systematic review and meta-analysis. PloS one. 2018;13(1):e0191633. doi:10.1371/journal.pone.0191633
27. Adams NL, Byrne L, Smith GA, et al. Shiga Toxin-Producing Escherichia coli O157, England and Wales, 1983-2012. Emerging Infectious Diseases. 2016;22(4):590-7. doi:10.3201/eid2204.151485
28. Tserenpuntsag B, Chang H-G, Smith PF, Morse DL. Hemolytic uremic syndrome risk and Escherichia coli O157:H7. Emerging Infectious Diseases. 2005;11(12):1955-7.
279 Bell BP, Griffin PM, Lozano P, Christie DL, Kobayashi JM, Tarr PI. Predictors of hemolytic uremic syndrome in children during a large outbreak of Escherichia coli O157:H7 infections. Pediatrics. 1997;100(1):E12.
30. Rowe PC, Orrbine E, Lior H, et al. Risk of hemolytic uremic syndrome after sporadic Escherichia coli O157:H7 infection: results of a Canadian collaborative study. Investigators of the Canadian Pediatric Kidney Disease Research Center. Journal of Pediatrics. 1998;132(5):777-82.
31. Cimolai N, Basalyga S, Mah DG, Morrison BJ, Carter JE. A continuing assessment of risk factors for the development of Escherichia coli O157:H7-associated hemolytic uremic syndrome. Clinical Nephrology. 1994;42(2):85-9.
32. Chang H-GH, Tserenpuntsag B, Kacica M, Smith PF, Morse DL. Hemolytic uremic syndrome incidence in New York. Emerging Infectious Diseases. 2004;10(5):928-31.

https://mc.manuscriptcentral.com/bmjpo

TABLES

Table 1: Characteristics of cohort participants by HUS status (n=1,059)

		No HUS<br>n (%)	HUS<br>n (%)
**Total**		852 (80.5)	207 (19.6)
**Age group**	<1	64 (91.4)	6 (8.6)
	1-4	370 (76.1)	116 (23.9)
	5-9	239 (80.7)	57 (19.3)
	10-15	179 (86.5)	28 (13.5)
**Sex**	Female	400 (77.5)	116 (22.5)
	Male	452 (83.2)	91 (16.8)
**Age and Sex**	Female <1	24 (85.7)	4 (14.3)
	Female 1-4	171 (74.0)	60 (26.0)
	Female 5-9	117 (79.1)	31 (20.9)
	Female 10-15	88 (80.7)	21 (19.3)
	Male <1	40 (95.2)	2 (4.8)
	Male 1-4	199 (78.0)	56 (22.0)
	Male 5-9	122 (82.4)	26 (17.6)
	Male 10-15	91 (92.9)	7 (7.1)
**Ethnicity**	White	552 (80.5)	134 (19.5)
	Non-white	138 (88.5)	18 (11.5)
	Unknown	162 (74.7)	55 (23.4)
**IMD Quintile**	1 (Least Disadvantaged)	198 (80.8)	47 (19.2)
	2	186 (84.2)	35 (15.8)
	3	166 (75.8)	53 (24.2)
	4	142 (76.8)	43 (23.2)
	5 (Most Disadvantaged)	160 (84.7)	29 (15.3)
**Travel**	Yes	128 (85.3)	22 (14.7)
	No	724 (79.7)	185 (20.4)
**Rurality**	Rural	230 (80.4)	56 (19.6)
	Urban	622 (80.5)	151 (19.5)
**Region**	East Midlands	65 (81.3)	15 (18.8)
	East of England	57 (80.3)	14 (19.7)
	London	93 (81.6)	21 (18.4)
	North East	64 (77.1)	19 (22.9)
	North West	153 (77.7)	44 (22.3)
	South East	92 (78.6)	25 (21.4)
	South West	101 (75.9)	32 (24.1)
	West Midlands	96 (84.2)	18 (15.8)
	Yorkshire and Humber	131 (87.3)	19 (12.7)
Stx	*Stx*1	17 (94.4)	1 (5.6)
	*Stx*2	609 (81.7)	136 (18.3)
	*Stx*1+2	219 (96.9)	7 (3.1)
	Serology	7 (10.6)	59 (89.4)
	Unknown	0 (0.0)	4 (100.0)
**Symptoms**	Diarrhoea	803 (80.3)	197 (19.7)
	Bloody diarrhoea	432 (74.0)	152 (26.0)
	Nausea	278 (75.8)	89 (24.3)
	Vomiting	330 (66.1)	169 (33.9)
	Abdominal pain	574 (78.2)	160 (21.8)

Confidential: For Review Only

	Fever	273 (76.7)	83 (23.3)
**Healthcare contact**	Antibiotics	53 (40.8)	77 (59.2)
	NHS Direct	67 (72.0)	26 (28.0)
	GP	570 (83.7)	111 (16.3)
	A&E	186 (66.9)	92 (33.1)
	Other healthcare contact	98 (74.8)	33 (25.2)
	Hospital	223 (52.4)	203 (47.6)

HUS – haemolytic uraemic syndrome; *stx* – Shiga toxin; NHS Direct – National Health Service telephone advice line, now NHS 111; GP – General Practitioner; A&E – accident and emergency

Confidential: For Review Only

Table 2: Adjusted and unadjusted regression analysis (n=989)

Variable	Category	n (%)	Unadjusted		Adjusted[a]		p value[b]
			Odds Ratio	(95% CI)	Odds Ratio	(95% CI)	
**Age group**	<1	67 (6.8)	0.19	(0.06-0.62)	**0.21**	**(0.05-0.82)**	**0.03**
	1-4	456 (46.1)	1.0 (reference)		1.0 (reference)		
	5-9	274 (27.7)	0.62	(0.40-0.94)	**0.43**	**(0.25-0.74)**	**0.002**
	10-15	192 (19.4)	0.34	(0.19-0.61)	**0.20**	**(0.09-0.43)**	**<0.001**
**Sex**	Male	513 (51.9)	1.0 (reference)		1.0 (reference)		
	Female	476 (48.1)	1.37	(0.96-1.96)	1.38	(0.88-2.14)	0.16
**Ethnicity**[±]	White	797 (80.6)	1.0 (reference)		1.0 (reference)		
	Non-White	192 (19.4)	0.39	(0.18-0.81)	**0.28**	**(0.11-0.74)**	**0.01**
**Travel**	No	850 (86.0)	1.0 (reference)		1.0 (reference)		
	Yes	139 (14.0)	0.46	(0.24-0.88)	0.64	(0.28-1.45)	0.28
**Rurality**	Urban	719 (72.7)	1.0 (reference)		1.0 (reference)		
	Rural	270 (27.3)	1.21	(0.82-1.77)	0.88	(0.52-1.48)	0.63
**IMD Quintile**	1 (least disadvantaged)	231 (23.4)	1.0 (reference)		1.0 (reference)		
	2	210 (21.2)	0.83	(0.48-1.42)	0.64	(0.32-1.27)	0.20
	3	204 (20.6)	1.28	(0.77-2.12)	1.01	(0.54-1.91)	0.97
	4	170 (17.2)	1.10	(0.64-1.90)	1.10	(0.54-2.26)	0.79
	5 (most disadvantaged)	174 (17.6)	0.57	(0.30-1.06)	0.57	(0.25-1.31)	0.18
**Region**	East Midlands	72 (7.3)	0.62	(0.24-1.59)	0.59	(0.18-1.92)	0.39
	East of England	66 (6.7)	1.03	(0.44-2.42)	1.12	(0.37-3.37)	0.84
	London	108 (10.9)	1.0 (reference)		1.0 (reference)		
	North East	76 (7.7)	1.19	(0.53-2.64)	0.71	(0.26-1.97)	0.51
	North West	185 (18.7)	1.20	(0.63-2.31)	1.02	(0.44-2.37)	0.97
	South East	107 (10.8)	1.09	(0.52-2.28)	1.31	(0.48-3.63)	0.60
	South West	127 (12.8)	1.48	(0.75-2.93)	1.25	(0.50-3.13)	0.63
	West Midlands	104 (10.5)	0.54	(0.23-1.29)	0.53	(0.18-1.53)	0.24
	Yorkshire and Humber	144 (14.6)	0.62	(0.29-1.33)	0.52	(0.20-1.34)	0.17
Stx	*Stx*1+2	226 (22.9)	1.0 (reference)		1.0 (reference)		
	*Stx*1	18 (1.8)	1.84	(0.21-15.84)	5.53	(0.53-57.42)	0.15
	*Stx*2	745 (75.3)	6.99	(3.22-15.17)	**5.92**	**(2.49-14.10)**	**<0.001**
**Antibiotics**	No	887 (89.7)	1.0 (reference)		1.0 (reference)		

Confidential: For Review Only

	Yes	102 (10.3)	8.54	(5.48-13.30)	**8.46**	**(4.71-15.18)**	**<0.001**
**Diarrhoea**	No	49 (5.0)	1.0 (reference)		1.0 (reference)		
	Yes	940 (95.0)	8.61	(1.18-62.89)	4.04	(0.50-32.59)	0.19
**Bloody diarrhoea**	No	440 (44.5)	1.0 (reference)		1.0 (reference)		
	Yes	549 (55.5)	4.85	(3.07-8.00)	**3.56**	**(2.04-6.24)**	**<0.001**
**Nausea**	No	653 (66.0)	1.0 (reference)		1.0 (reference)		
	Yes	336 (34.0)	1.52	(1.06-2.18)	1.12	(0.67-1.86)	0.66
**Vomiting**	No	549 (55.5)	1.0 (reference)		1.0 (reference)		
	Yes	440 (44.5)	6.05	(3.95-9.26)	**4.47**	**(2.62-7.63)**	**<0.001**
**Abdominal pain**	No	309 (31.2)	1.0 (reference)		1.0 (reference)		
	Yes	680 (68.8)	1.49	(0.99-2.25)	0.82	(0.46-1.46)	0.50
**Fever**	No	657 (66.4)	1.0 (reference)		1.0 (reference)		
	Yes	332 (33.6)	1.50	(1.05-2.16)	1.05	(0.67-1.66)	0.82

[a]Adjusted for all other covariates in the model; [b]Statistical significance of relationship between HUS and each variable tested using χ^2 test ; *stx* – Shiga toxin gene; [±]Multiply imputed variable

Confidential: For Review Only

SUPPLEMENTARY DATA

Supplementary Table 1a: Clinical case definition for HUS

A child (aged <16 years of age) who has:
Acute kidney injury (AKI) defined by oligoanuria and/or elevated creatinine for age
AND
Microangiopathic haemolytic anamia (MAHA) defined by haemoglobin level <10 g/L with fragmented erythrocytes
AND/OR
Thrombocytopenia defined by a platelet count of <130,000 × 109/L
WITHOUT septicaemia, malignant hypertension, chronic uraemia, or primary vascular disease
Elevated creatinine levels differed by age group and were those above the thresholds in Table 1

Supplementary Table 1b: Creatinine level (micromol/L) thresholds by age group

**Age Group**	**Normal Creatinine threshold (micromol/L)**
0-7 days	100
8-14 days	80
15-28 days	55
1m-3 years	40
4-6 years	46
7-9 years	56
10-12 years	60
13-15 years	80

Confidential: For Review Only

Supplementary Table 2: Adjusted and unadjusted regression analysis - Sensitivity analysis excluding travel cases (n subjects=850)

Variable	Category	n (%)	Unadjusted		Adjusted[a]		p value
			Odds Ratio	(95% CI)	Odds Ratio	(95% CI)	
**Age group**	<1	55 (6.5)	0.14	(0.03-0.59)	**0.12**	**(0.02-0.62)**	**0.01**
	1-4	400 (47.1)	1.0 (reference)		1.0 (reference)		
	5-9	242 (28.5)	0.61	(0.39-0.94)	**0.45**	**(0.25-0.79)**	**0.005**
	10-15	153 (18.0)	0.32	(0.17-0.60)	**0.17**	**(0.07-0.39)**	**<0.001**
**Sex**	Male	445 (52.4)	1.0 (reference)		1.0 (reference)		
	Female	405 (47.7)	1.31	(0.91-1.90)	1.30	(0.81-2.08)	0.27
**Ethnicity**	White	700 (82.4)	1.0 (reference)		1.0 (reference)		
	Non-White	150 (17.6)	0.41	(0.20-0.83)	**0.27**	**(0.10-0.73)**	**0.01**
**Rurality**	Urban	606 (71.3)	1.0 (reference)		1.0 (reference)		
	Rural	244 (28.7)	1.28	(0.86-1.90)	1.07	(0.62-1.86)	0.80
**IMD Quintile**	1 (least disadvantaged)	196 (23.1)	1.0 (reference)		1.0 (reference)		
	2	184 (21.7)	0.88	(0.50-1.54)	0.75	(0.36-1.53)	0.43
	3	180 (21.2)	1.24	(0.73-2.10)	1.06	(0.54-2.10)	0.86
	4	150 (17.7)	1.07	(0.61-1.90)	1.14	(0.53-2.46)	0.74
	5 (most disadvantaged)	140 (16.5)	0.52	(0.26-1.04)	0.57	(0.23-1.43)	0.23
**Region**	East Midlands	61 (7.2)	0.54	(0.19-1.49)	0.49	(0.14-1.80)	0.29
	East of England	55 (6.5)	0.84	(0.33-2.16)	1.02	(0.30-3.44)	0.98
	London	83 (9.8)	1.0 (reference)		1.0 (reference)		
	North East	71 (8.4)	0.90	(0.38-2.14)	0.59	(0.19-1.81)	0.35
	North West	172 (20.2)	1.13	(0.56-2.25)	1.06	(0.42-2.67)	0.90
	South East	73 (8.6)	1.27	(0.58-2.86)	1.78	(0.57-5.56)	0.32
	South West	110 (12.9)	1.38	(0.66-2.86)	1.14	(0.42-3.11)	0.80
	West Midlands	95 (11.2)	0.52	(0.21-1.26)	0.57	(0.18-1.78)	0.33
	Yorkshire and Humber	130 (15.3)	0.59	(0.27-1.32)	0.53	(0.19-1.47)	0.22
Stx	*Stx*1+2	183 (21.5)	1.0 (reference)		1.0 (reference)		
	*Stx*1	8 (0.9)	4.21	(0.45-39.89)	**24.71**	**(1.86-328.34)**	**0.02**
	*Stx*2	659 (77.5)	6.97	(3.02-16.10)	**6.08**	**(2.32-15.92)**	**<0.001**
**Antibiotics**	No	766 (90.1)	1.0 (reference)		1.0 (reference)		
	Yes	84 (9.9)	10.0	(6.18-16.32)	**10.89**	**(5.65-20.97)**	**<0.001**
**Diarrhea**	No	48 (6.6)	1.0 (reference)		1.0 (reference)		
	Yes	802 (94.4)	9.26	(1.27-67.70)	4.00	(0.48-33.16)	0.20

Confidential: For Review Only

**Bloody diarrhea**	No	360 (42.4)	1.0 (reference)		1.0 (reference)		
	Yes	490 (57.7)	5.10	(3.10-8.38)	**3.70**	**(2.00-6.85)**	**<0.001**
**Nausea**	No	562 (66.1)	1.0 (reference)		1.0 (reference)		
	Yes	288 (33.9)	1.52	(1.04-2.22)	1.03	(0.60-1.76)	0.92
**Vomiting**	No	465 (54.7)	1.0 (reference)		1.0 (reference)		
	Yes	385 (45.3)	6.50	(4.13-10.23)	**5.25**	**(2.94-9.38)**	**<0.001**
**Abdominal pain**	No	259 (30.5)	1.0 (reference)		1.0 (reference)		
	Yes	591 (69.5)	1.39	(0.91-2.13)	0.73	(0.39-1.36)	0.32
**Fever**	No	569 (66.9)	1.0 (reference)		1.0 (reference)		
	Yes	281 (33.1)	1.71	(1.17-2.50)	1.28	(0.79-2.09)	0.32

aAdjusted for all other covariates in the model; *stx* – shiga toxin gene

Confidential: For Review Only

Supplementary Table 3: Adjusted and unadjusted regression analysis - Sensitivity analysis excluding ethnicity variable (n subjects=989)

Variable	Category	n (%)	Unadjusted		Adjusted[a]		p value
			Odds Ratio	(95% CI)	Odds Ratio	(95% CI)	
**Age group**	<1	67 (6.8)	0.19	(0.06-0.62)	**0.24**	**(0.06-0.92)**	**0.04**
	1-4	456 (46.1)	1.0 (reference)		1.0 (reference)		
	5-9	274 (27.7)	0.62	(0.40-0.94)	**0.45**	**(0.26-0.76)**	**0.003**
	10-15	192 (19.4)	0.34	(0.19-0.61)	**0.22**	**(0.11-0.47)**	**<0.001**
**Sex**	Male	513 (51.9)	1.0 (reference)		1.0 (reference)		
	Female	476 (48.1)	1.37	(0.96-1.96)	1.40	(0.91-2.16)	0.13
**Travel**	No	850 (86.0)	1.0 (reference)		1.0 (reference)		
	Yes	139 (14.0)	0.46	(0.24-0.88)	0.58	(0.26-1.28)	0.18
**Rurality**	Urban	719 (72.7)	1.0 (reference)		1.0 (reference)		
	Rural	270 (27.3)	1.21	(0.82-1.77)	0.97	(0.58-1.62)	0.90
**IMD Quintile**	1 (least disadvantaged)	231 (23.4)	1.0 (reference)		1.0 (reference)		
	2	210 (21.2)	0.83	(0.48-1.42)	0.65	(0.33-1.27)	0.21
	3	204 (20.6)	1.28	(0.77-2.12)	0.98	(0.51-1.79)	0.89
	4	170 (17.2)	1.10	(0.64-1.89)	0.96	(0.48-1.94)	0.91
	5 (most disadvantaged)	174 (17.6)	0.57	(0.30-1.06)	**0.41**	**(0.19-0.90)**	**0.03**
**Region**	East Midlands	72 (7.3)	0.62	(0.24-1.59)	0.65	(0.21-2.02)	0.45
	East of England	66 (6.7)	1.03	(0.44-2.42)	1.37	(0.47-4.00)	0.56
	London	108 (10.9)	1.0 (reference)		1.0 (reference)		
	North East	76 (7.7)	1.19	(0.53-2.64)	1.02	(0.38-2.71)	0.97
	North West	185 (18.7)	1.20	(0.63-2.31)	1.29	(0.57-2.90)	0.54
	South East	107 (10.8)	1.09	(0.52-2.28)	1.74	(0.65-4.63)	0.27
	South West	127 (12.8)	1.48	(0.75-2.93)	1.64	(0.68-3.99)	0.27
	West Midlands	104 (10.5)	0.54	(0.23-1.29)	0.67	(0.24-1.86)	0.44
	Yorkshire and Humber	144 (14.6)	0.62	(0.29-1.33)	0.60	(0.24-1.52)	0.28
Stx	*Stx*1+2	226 (22.9)	1.0 (reference)		1.0 (reference)		
	*Stx*1	18 (1.8)	1.84	(0.21-15.84)	5.34	(0.54-52.82)	0.15
	*Stx*2	745 (75.3)	6.99	(3.22-15.17)	**5.76**	**(2.43-13.67)**	**<0.001**
**Antibiotics**	No	887 (89.7)	1.0 (reference)		1.0 (reference)		
	Yes	102 (10.3)	8.54	(5.48-13.30)	**7.46**	**(4.27-13.03)**	**<0.001**
**Diarrhea**	No	49 (5.0)	1.0 (reference)		1.0 (reference)		
	Yes	940 (95.0)	8.61	(1.18-62.89)	4.09	(0.51-32.47)	0.18

Confidential: For Review Only

**Bloody diarrhea**	No	440 (44.5)	1.0 (reference)		1.0 (reference)		
	Yes	549 (55.5)	4.85	(3.07-7.67)	**3.74**	**(2.15-6.49)**	**<0.001**
**Nausea**	No	653 (66.0)	1.0 (reference)		1.0 (reference)		
	Yes	336 (34.0)	1.52	(1.06-2.18)	1.11	(0.67-1.83)	0.69
**Vomiting**	No	549 (55.5)	1.0 (reference)		1.0 (reference)		
	Yes	440 (44.5)	6.05	(3.95-9.26)	**4.38**	**(2.59-7.40)**	**<0.001**
**Abdominal pain**	No	309 (31.2)	1.0 (reference)		1.0 (reference)		
	Yes	680 (68.8)	1.49	(0.99-2.25)	0.83	(0.47-1.46)	0.52
**Fever**	No	657 (66.4)	1.0 (reference)		1.0 (reference)		
	Yes	332 (33.6)	1.50	(1.05-2.16)	1.04	(0.66-1.63)	0.86

aAdjusted for all other covariates in the model; *stx* – shiga toxin gene

Confidential: For Review Only

Supplementary Figure 1: Selection of participants to HUS Cohort Study

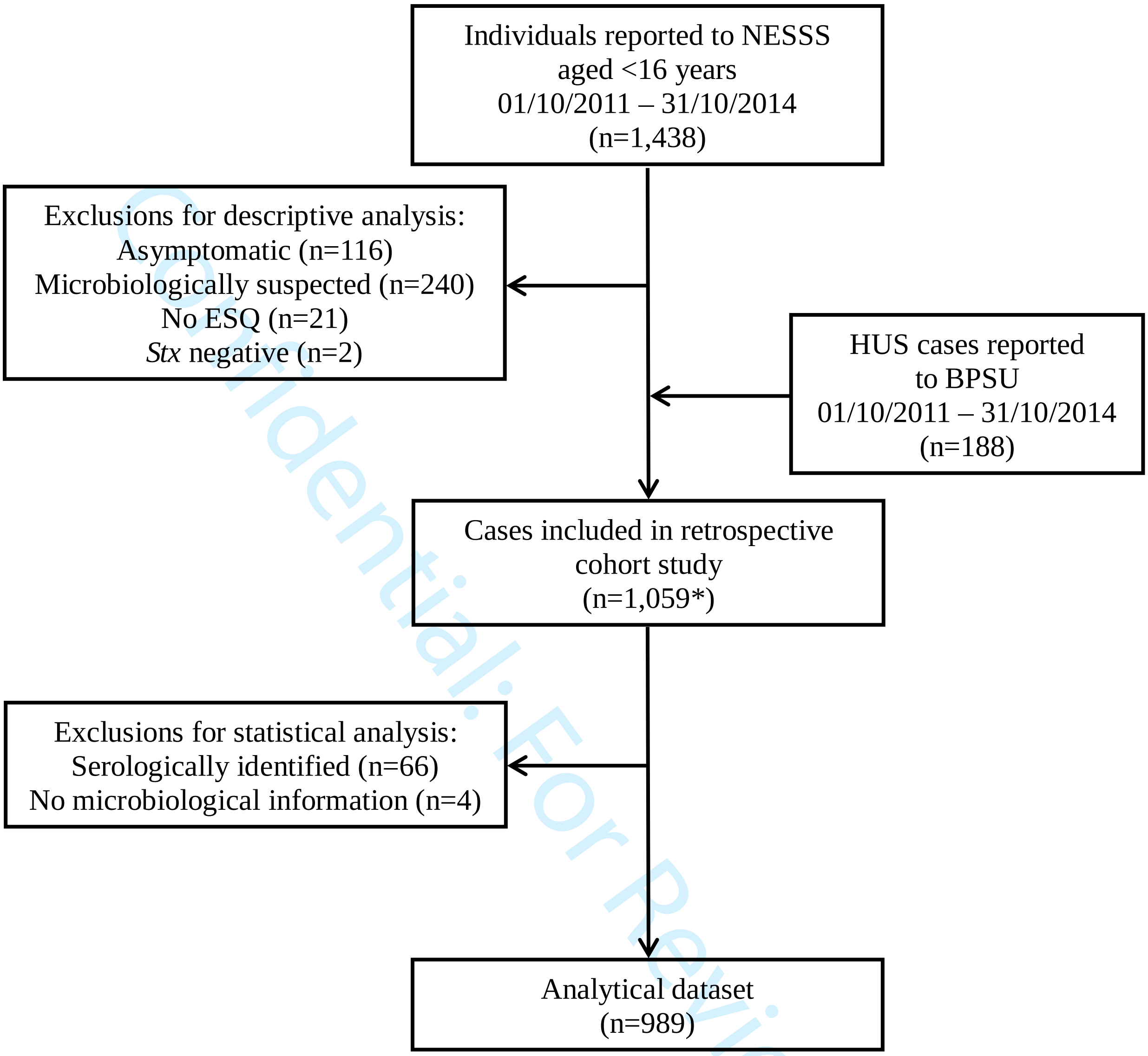

*An additional 19 HUS cases not reported to BPSU were identified in NESSS; NESSS – National Enhanced Surveillance System for STEC; HUS – haemolytic uraemic syndrome; BPSU – British Paediatric Surveillance Unit; ESQ – enhanced surveillance questionnaire

Confidential: For Review Only
